# Intuitive Sociology: Children Recognize Decision-Making Structures and Prefer Groups With Less-Concentrated Power

**DOI:** 10.1162/opmi_a_00053

**Published:** 2022-07-01

**Authors:** Ashley J. Thomas, Vivian Mitchell, Emily Sumner, Brandon F. Terrizzi, Paul K. Piff, Barbara W. Sarnecka

**Affiliations:** Brain and Cognitive Sciences, Massachusetts Institute of Technology; Department of Psychological Science, University of California, Irvine; Cognitive Sciences, University of California, Irvine; Division of General and Community Pediatrics, Cincinnati Children’s Hospital Medical Center

**Keywords:** social reasoning, social development, social hierarchy, participatory decision-making

## Abstract

From an early age, children recognize that people belong to social groups. However, not all groups are structured in the same way. The current study asked whether children recognize and distinguish among different decision-making structures. If so, do they prefer some decision-making structures over others? In these studies, children were told stories about two groups that went camping. In the hierarchical group, one character made all the decisions; in the egalitarian group, each group member made one decision. Without being given explicit information about the group’s structures, 6- to 8-year-old children, but not 4- and 5-year-old children, recognized that the two groups had different decision-making structures and preferred to interact with the group where decision-making was shared. Children also inferred that a new member of the egalitarian group would be more generous than a new member of the hierarchical group. Thus, from an early age, children’s social reasoning includes the ability to compare social structures, which may be foundational for later complex political and moral reasoning.

## INTRODUCTION

Humans are unlike other social species in that they can recognize the social structures they live in, imagine alternatives, and act to change these structures. However, little is known about the development of these abilities. Here we ask whether children, ages 4 to 8 years old, recognize that groups can organize decision-making in different ways. Previous research on children’s understanding of social groups has found that children reason about ingroups and outgroups. Other work has shown that children recognize social roles in dyads (e.g., “being in charge”). The current studies show that children, ages 6 to 8 years, also reason about the structures of social groups. In these studies, children guessed that members of a group that shared decision-making would also share resources, and preferred egalitarian groups to hierarchical ones.

Imagine you are a child who just started at a new school. During recess, you notice two groups of children. In the first group, one person makes all the decisions: she decides where the group sits for lunch, what snacks they eat, and which games they play. In the second group, the children take turns making decisions—one child decides where they sit, another child decides what snacks they eat, and a third child decides which games they play. In order to successfully interact with these groups, you would rely on your “intuitive sociology”—your understanding of how social relationships and social groups work (Hirschfeld, 2001; Kaufmann & Clément, 2014). Intuitive sociology would allow you to identify which individuals belong to which group and the structures of the two groups, which in turn may inform your predictions about how the people in the two groups will behave, as well as your decision about which group to approach. Intuitive sociology is particularly important because we live in complex social worlds that can be structured in different ways (Keltner et al., 2008; Wengrow & Graeber, 2015). This creates a particular challenge for children, who need to eventually understand how their social world is divided, and how different groups are structured (Fiske, 1992; Kemp & Tenenbaum, 2008; Thomsen & Carey, 2013). For example, an employee who fails to recognize that their workplace is organized hierarchically may lose their job. On the other hand, many other groups place a high value on egalitarianism. For example, in many hunter-gatherer societies, trying to assume too much power can lead to ostracism and in some cases, even death (Boehm, 2001; Wrangham, 2019). Finally, being able to recognize the structure of one’s social world, as well as imagine alternative arrangements, is also necessary for societal change. For example, social movements are driven by people who both recognize the social structures in which they live and imagine alternatives.

Although we do not know much about children’s understanding of within-group structures, we do know that children have expectations about how people will treat ingroup members compared to outgroup members. Children expect members of ingroups to be similar to one another, to be morally obligated to one another, and to direct antisocial actions toward outgroup members (see Chalik et al., 2019, for review). These studies have been taken as evidence that children possess an intuitive theory of social groups (i.e., an abstract, domain-specific, causal explanatory framework (Chalik & Dunham, 2020). Strikingly, these inferences are often the result of children reasoning about minimal groups (groups where membership is based on an arbitrary assignment such as shirt color). When it comes to social groups based on identities such as race and gender, children not only see “ingroups” and “outgroups” but also relative status between social groups (Mandalaywala et al., 2020). Thus, seeing “us” and “them” has been argued to be a fundamental aspect of our social cognition (Dunham, 2018). However, another way to think about groups, including one’s social groups, is to recognize their internal structure. Do children also develop the ability to recognize and compare different social structures? Do children use these inferences to evaluate groups they encounter? Are children’s ideas about group structure theory-like in that they use group structure to infer how people will behave across contexts?

One reason to think that young children recognize the social structures of groups comes from studies showing that children recognize social roles. For example, preschool-age children say that a person who adopts an expansive posture toward someone with a constricted posture is “in charge” (Brey & Shutts, 2015; Terrizzi et al., 2018). By age 4, children say that those who control resources, give permission, and are deferred to in a conflict are “in charge” relative to the targets of those actions (Gülgöz & Gelman, 2017). Toddlers and children also evaluate others based on their role in a dyadic relationship. For example, toddlers 21 to 31 months reach for the winners of zero-sum conflicts (Thomas et al., 2018) and children aged 3 to 7 years say they prefer high-ranking individuals across a variety of contexts (Charafeddine et al., 2016; Enright et al., 2020). These studies suggest that children identify relative social rank within dyadic interactions and prefer those who are higher ranked.

But when do children recognize that the pair could relate to one another in a different way, for example, having *no one* in charge? Moreover, a desire to affiliate with a higher-ranked individual does not necessarily mean a desire to be in a social group in which rank exists. Being in a hierarchical setting may mean it is in one’s best interest to affiliate with higher-ranking individuals who can, for example, have more resources or can offer protection. However, one might still prefer to be in egalitarian groups where power is more equally distributed.

Children do seem to prefer equal distribution of resources. When all else is equal, children prefer to allocate equally (Baumard et al., 2012) and will even discard a resource to ensure that two other children get the same amounts (Shaw & Olson, 2012). Moreover, 5-year-old children can discover turn-taking as a cooperative strategy when it leads to rewards (Melis et al., 2016). However, children do not always prefer equal distributions: children are willing to allocate resources unequally when told that one of the target characters worked harder (e.g., Shaw & Olson, 2012), suggesting that children’s preferences for resource distribution depend on context (see also McAuliffe et al., 2017, for review). However, preferring equal distribution of resources does not tell us whether children prefer equal distribution of decision-making. For example, one could prefer that members of a group get equal resources, but that decision-making is concentrated to facilitate social coordination.

In the present studies, we investigated whether children’s ideas about social groups go beyond recognizing group membership. Do children track patterns of decision-making and infer attributes of the group? Across two studies, we examined when children, in American suburban/urban environments, recognize that social groups can be structured in different ways. We investigated whether children’s ideas about social structure are theory-like, by asking whether they use one aspect of a group (decision-making structure) to predict another aspect (resource distribution). In Study 1 we investigate this last question by asking, “Who shares more?” and in Study 2 we investigate this last question by asking children to predict the number of resources a new group member will share with another member of their group. Finally, we asked whether children preferred one type of social structure to another.

## GENERAL METHODS

In both studies, children were introduced to two novel groups of three individuals each (“Wugs” and “Flurps”) who wore different-colored shirts. Both groups went camping and needed to make three decisions: Where to pitch the tent, what game to play, and what song to sing. In the hierarchical group, one character made all three decisions for the group, whereas in the egalitarian group, each of the three group members made one decision. In Study 1 we asked the children to identify which group had someone in charge, which group shared more, and which group the child preferred. In Study 2 we used open-ended questions to ask whether children spontaneously noticed the difference in decision-making between the two groups and again asked whether the children preferred either group. In Study 2 we also asked children to predict how many resources a new member of the group (who was not in the story about camping) would share with a groupmate. In both studies, children were not given explicit information about the structure of the group, thus, we tested whether children inferred the underlying social structure by observing the pattern by which the group made decisions.

## STUDY 1

### Participants

A total of 176 children, ages 4 to 8 years, participated in Study 1. We chose this age range because prior work on ingroup and outgroup biases used a similar age range (Chalik & Rhodes, 2020; Mandalaywala, 2020). Of the initial 176 children, five children were excluded because of interference from siblings, and one was excluded because of experimenter error (the experimenter went off script), leaving 170 children in the sample. These were grouped by age into 4-year-olds (*n* = 42), 5-year-olds (*n* = 36), 6-year-olds (*n* = 35), 7-year-olds (*n* = 31), and 8-year-olds (*n* = 26). Our aim was to collect 25 subjects in each age range. Since we were testing at a museum, experimenters could not always accurately predict the age of children before approaching their parents, so we tested some children in each age range after reaching this goal. When asked about gender, 82 parents indicated their child was a boy, 84 indicated their child was a girl, and four didn’t answer the question. When asked to indicate racial background, 83 parents answered “White,” 32 did not answer the question, 23 answered “Asian,” 9 answered “African American,” 6 answered, “Asian and White,” 4 answered “American Indian/Alaska Native and White,” 4 answered “Asian and Native Hawaiian”; 9 indicated they were multiracial.

### Procedure

**Figure 1.**
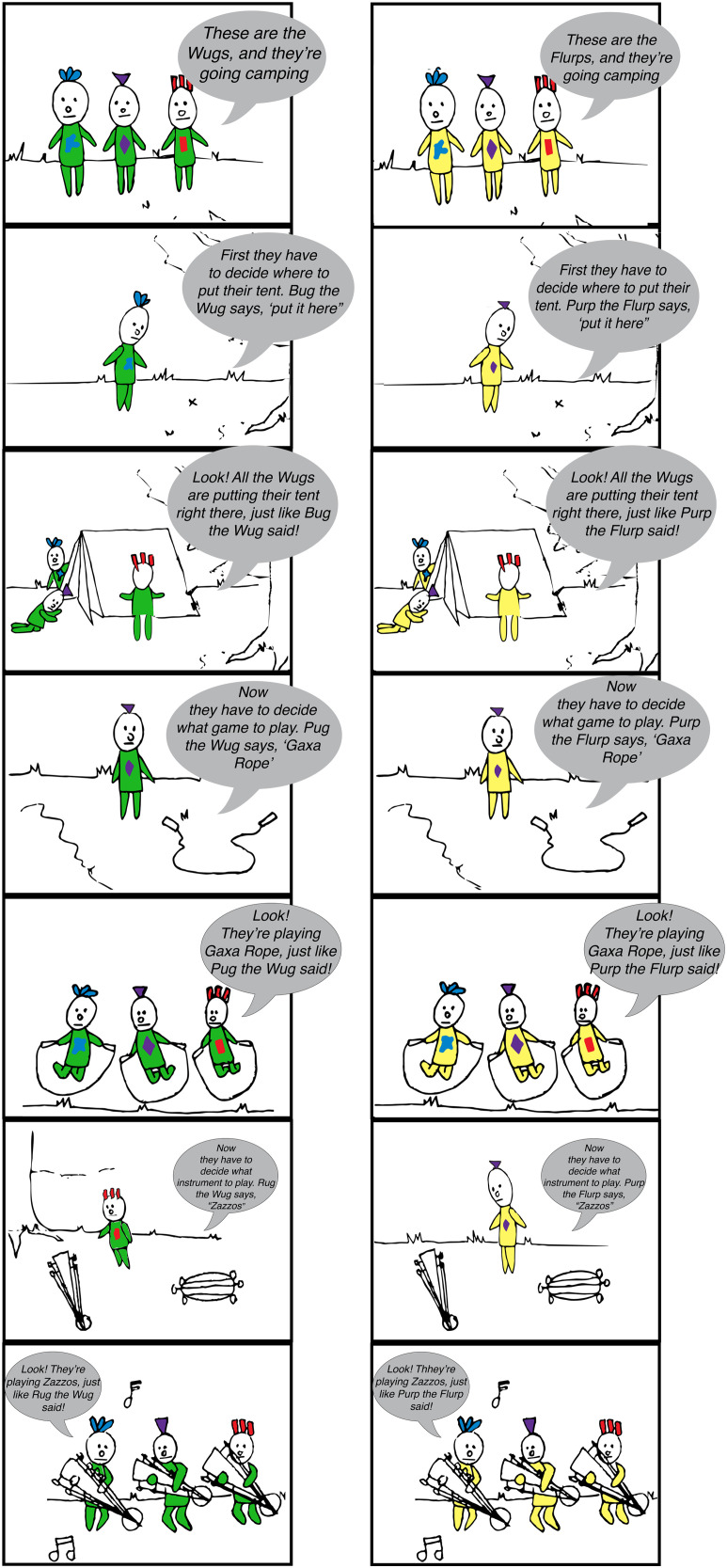
**Stimuli used in experiment.** [Left Panel] Egalitarian story: pictures and script text. [Right Panel] Hierarchical story: pictures and script text.

Children were recruited at a children’s museum. Parents were approached and asked if they wanted to participate in a study about children’s understanding of social relationships. If they agreed, the experimenter led the child and parent to a room off the main floor of the museum, where the parent filled out a consent form. The testing room had glass walls, so parents could watch from outside the room. In the experiment, children heard stories accompanied by pictures (see Figure 1).

Children were told two stories about two groups (the Wugs and the Flurps) who were going camping. In each story, three decisions were made in each group (e.g., “*First, they have to decide where to put the tent. Grug the Wug says, put it under the tree. Look! All the Wugs are setting up the tent under the tree, just like Grug the Wug said*”). In the hierarchical group, one character made all three decisions. In the egalitarian group, a different character made each decision. The pictures that accompanied the stories were laid on the table in chronological order. After hearing both stories, children were asked three questions (always in the same order): “*Would you rather be a Wug or a Flurp?*” “*Who would you rather go camping with?*” and “*Who do you think shares more with each other?*” Then, to check whether children differentiated between the two groups we asked, “*Which ones had someone who is in charge? Which ones had a boss?*” Then we asked, “*Who is the boss?*” or “*Who is in charge?*” Finally, we asked, “*Which ones took turns?*” (see the Supplementary Materials for analysis for the last two questions).

The names of the hierarchical and egalitarian groups were counterbalanced across participants. Half of the children heard about the hierarchical group first and half heard about the egalitarian group first. All scripts, stimuli, and data can be found here at https://osf.io/s6cu9/.

### Analysis Approach

For each of the main test questions, we asked whether age (in years), gender, and the order that the children heard about the groups predicted children’s answers to the dependent measure questions. To do this, we used the R package brms (Bürkner, 2017) in R (R Core Team, 2017).

To ask how these factors influenced children’s answers, we implemented the ROPE (region of practical equivalence) method using the package BayestestR (Makowski et al., 2019). To implement the ROPE method, one first establishes a range of values deemed consistent with the null hypothesis (Kruschke & Vanpaemel, 2015). We defined this null region as going from −0.1 to 0.1. We preregistered this analysis after we looked at results from another analysis that we realized was flawed. We preregistered that we would consider values of less than 2.5% to be evidence for the alternative hypothesis (Makowski et al., 2019; see Table 1). To calculate whether children’s responses differed from chance, we used a two-sided binomial test for each age group and calculated a Bayes Factor comparing the likelihood of the data arising under the null hypothesis (that children chose each group 50% of the time) with the likelihood of the data arising under the alternative hypothesis (that children chose one group or the other more than 50% of the time; Morey et al., 2014). An R-markdown file can be found in the Supplemental Materials, which shows plots of the posterior distributions for each model, and the ROPE overlaid on the plot. There are additional analyses in this file, including those that ask how children’s answers to the dependent measure questions were related and analyses of two dependent variables that are not included here.

**Table 1.** Output of Region of Practical Equivalence (ROPE) Analysis, Which Computes the Proportion of the Posterior Distribution That Lies Inside a Null Region

**Outcome Variable**	**Age**	**Gender**	**Order**
***Which ones had someone in charge?***	ROPE: 0.35%*	ROPE: 22.56%	ROPE: 21.85%
	Est: 0.45	Est: −0.01	Est: −0.04
	CI: 0.19 to 0.72	CI: −0.70 to 0.68	CI: −0.73 to 0.65
***Who do you think shares more with one another?***	ROPE: 0.58%*	ROPE: 22.57%	ROPE: 1.87%*
	Est: 0.41	Est: 0.08	Est: −0.77
	CI: 0.17 to 0.66	CI: −0.58 to 0.74	CI: 0.35 to −1.46
***Would you rather be a Wug or a Flurp?***	ROPE: 2.72%	ROPE: 22.44%	ROPE: 14.58%
	Est: −0.32	Est: 0.13	Est: −0.33
	CI: −0.56 to −0.10	CI: −0.56 to −0.10	CI: −0.96 to 0.30
***Who would you rather go camping with?***	ROPE: 1.10%*	ROPE: 8.54%	ROPE: 7.52%
	Est: −0.37	Est: −0.48	Est: −0.51
	CI: 0.12 to −0.61	CI: 0.33 to −1.13	CI: 0.33 to −1.15

*Note*. We defined that region as going from −.1 to .1. We preregistered this analysis after realizing that our original analysis approach was flawed, therefore we had run other analyses on this data before this analysis was carried out. We preregistered that we would consider outcomes of 2.5% or lower to be evidence for the alternative hypothesis that the factor had an effect (Makowski et al., 2019), marked with an * in the table. CI = confidence interval; Est = estimate.

### Results and Discussion

#### Which ones had someone “in charge?”

When we asked children, “*Which ones had someone in charge?*” 119/170 (69.82%) of children chose the hierarchical group. Older children were more likely than younger children to do so (ROPE = 0.35%). We did not find evidence that gender affected children’s answers (ROPE = 22.5%) nor that the order in which children heard the stories influenced their answers (ROPE = 21.85%).

**Figure 2.**
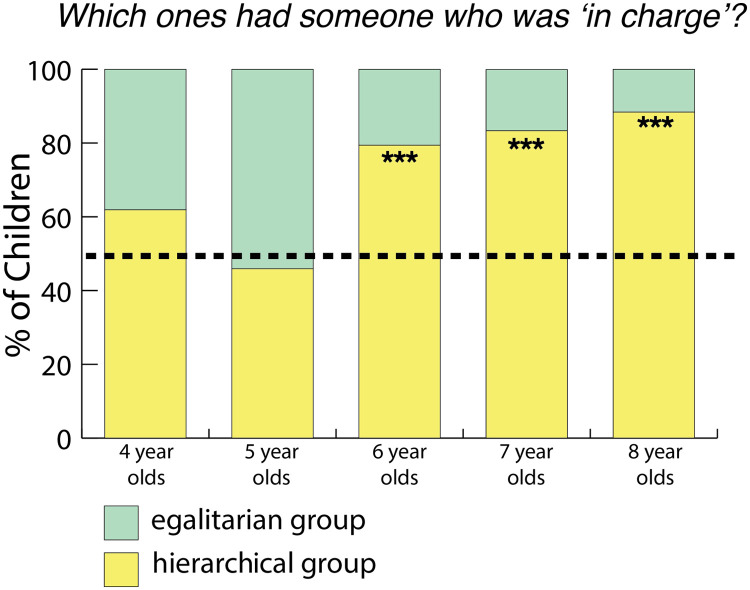
***“Which ones have someone in charge?”*** Percentage of children who chose the hierarchical group when asked, “*Which ones have someone in charge?*” in Study 1 (chance is 1/2). *** means strong evidence. Six- to 8-year-old children chose the hierarchical group (27/42 4-year-olds chose the hierarchical group, BF = 1.61; 16/36 5-year-olds chose the hierarchical group, BF = 0.46; 27/35 6-year-olds chose the hierarchical group, BF = 38; 26/31 7-year-olds chose the hierarchical group, BF = 249; 23/26 8-year-olds chose the hierarchical group, BF = 440. Bayes factors were calculated using a two-sided Bayesian binomial test as described in “Analysis Approach”).

When we broke down the children’s answers by year, only older children (6- to 8-year-olds) identified the hierarchical group as having someone “*in charge*” (see Figure 2). It is unclear from these data whether the task was too difficult for the younger children, or if they do not yet know that groups can be structured in different ways.

#### Which group shares more?

**Figure 3.**
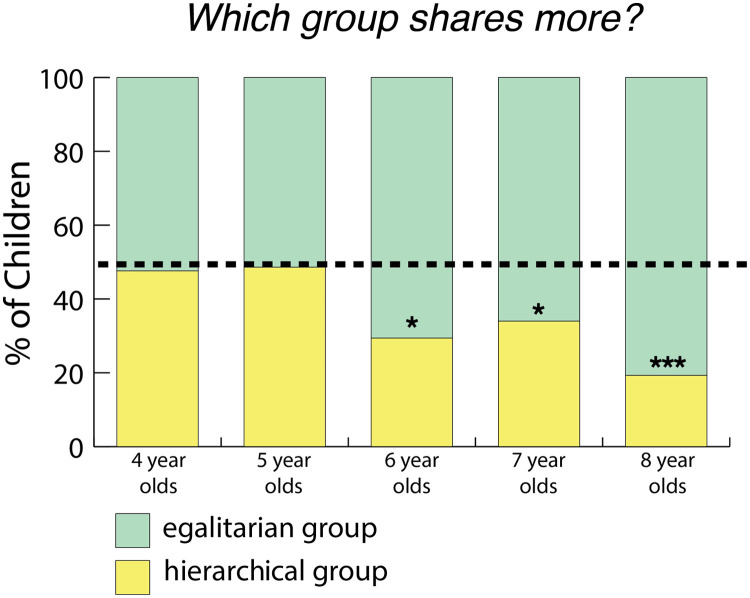
**Percentage of children in each age group choosing the hierarchical group when asked, “*Who shares more?*” in Study 1 (chance is 1/2).** *** means strong evidence; * means moderate evidence. Six- to 8-year-old children chose the egalitarian group (19/42 4-year-olds chose the egalitarian group, BF = 0.42; 19/36 5-year-olds chose the egalitarian group, BF = 0.399; 25/35 6-year-olds chose the egalitarian group, BF = 6.34; 21/31 7-year-olds chose the egalitarian group, BF = 2.11; 21/26 8-year-olds chose the egalitarian group, BF = 29.96. Bayes factors were calculated using a two-sided Bayesian binomial test as described in “Analysis Approach”).

When asked, “*Who shares more?*” children were more likely to choose the egalitarian group: 105/170 (62.17%) of the children chose the egalitarian group (BF = 18.11 in favor of the alternative). Older children were more likely than younger children to do so (ROPE = 0.58%). We did not find evidence that gender affected children’s answers (ROPE = 22.57%), but we did find evidence that the order that children heard the stories affected their answers (ROPE = 1.87%; see the Supplemental Materials for more detailed analysis). Only 6- to 8-year-olds chose the egalitarian group more often (see Figure 3).

These findings suggest that older children generalized from decision-making patterns to another attribute of the group—sharing. However, it is unclear how the children interpreted this question since we did not specify what we meant by sharing. Moreover, the order effect suggests that children may have been biased to say that the group they last heard about shares more with one another. In Study 2 we directly investigate whether children expect more sharing from egalitarian groups by asking children to predict the number of resources a member of each group will share with another member of their group.

#### Would you rather be a Wug or a Flurp?

**Figure 4.**
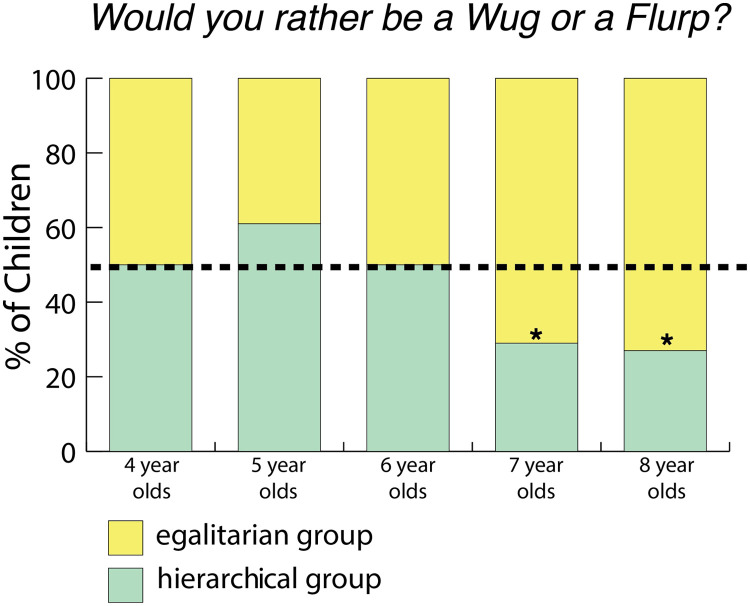
**Percentage of children who chose the hierarchical group when asked, “*Would you rather be a Wug or a Flurp?”* in Study 1 (chance is 1/2).** * indicates moderate evidence. Seven- and 8-year-olds chose the egalitarian group (21/42 4-year-olds chose the egalitarian group, BF = 0.357; 14/36 5-year-olds chose the egalitarian group, BF = 0.814; 18/35 6-year-olds chose the egalitarian group, BF = 0.39; 22/31 7-year-olds chose the egalitarian group, BF = 4.15; 19/26 8-year-olds chose the egalitarian group, BF = 4.36. Bayes factors were calculated using a two-sided Bayesian binomial test as described in “Analysis Approach”).

When asked, “*Would you Rather be a Wug or a Flurp?*” we found that children chose both groups equally often: 94/170 (57.99%) of children chose the egalitarian group (BF = 2.12 in favor of the null). Older children were more likely than younger children to do so (ROPE = 2.48%). We did not find evidence that gender affected children’s answers (ROPE = 22.4%) nor the order in which children heard the stories (ROPE = 14.58%). Only 7- and 8-year-olds chose one group more than the other: 7- and 8-year-olds chose the egalitarian group (see Figure 4).

#### Who would you rather go camping with?

**Figure 5.**
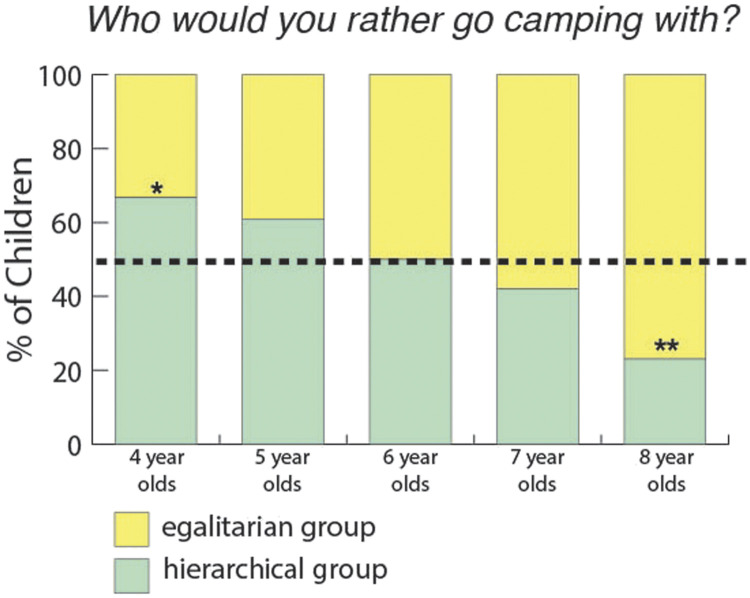
**Percentage of children who chose the hierarchical group when asked, “*Who would you rather go camping with?”* in Study 1 (chance is 1/2).** * indicates moderate evidence, ** means strong evidence. We found positive evidence that 8-year-olds and 4-year-olds had a preference (14/42 4-year-olds chose the egalitarian group, BF = 2.79; 22/36 5-year-olds chose the egalitarian group, BF = 0.814; 18/35 6-year-olds chose the egalitarian group, BF = 0.39; 18/31 7-year-olds chose the egalitarian group, BF = 0.566; 20/26 8-year-olds chose the egalitarian group, BF = 10.51. Bayes factors were calculated using a two-sided Bayesian binomial test as described in “Analysis Approach”).

When we asked children, “*Who would you rather go camping with?*” children chose both groups equally often: 92/170 (54%) of the children chose the egalitarian group, BF = 3.05 in favor of the null). Older children were more likely than younger children to do so (ROPE = 1.10%). We did not find evidence that gender affected children’s answers (ROPE = 8.54%) nor the order in which children heard the stories (ROPE = 7.52%). When we analyzed the children’s responses by age group, only 8-year-olds and 4-year-olds had a preference (see Figure 5).

These results suggest that by the age of 6 years, children are able to distinguish between two types of decision-making patterns and use those decision-making patterns to generalize to other behavior (sharing). Eight-year-olds, and to a lesser extent, 7-year-olds, also incorporated this distinction into their own decisions about which groups to interact with. The inferences of 4- and 5-year-olds are less clear: they failed to correctly identify the group that had a leader, thus, it is likely that when they were asked to choose a group to go camping with or a group to join, they did not incorporate the decision-making patterns into their answers. We collected data for a subsequent study (included in the Supplemental Materials) in which we repeated this experiment but told the children whether the group had someone in charge when we introduced the groups (e.g., “*These are the Wugs, they have someone in charge*”). Five- and 6-year-old children, but not 4-year-old children, were able to remember this information, but chose the two groups equally often when asked about which group they preferred (see the Supplemental Materials for more details).

## STUDY 2

Study 2 was designed to be a stronger test of the hypothesis that children’s reasoning about the structure of social groups is theory-like. As in Study 1, in Study 2 children first heard the story about the two groups. Unlike in Study 1, in Study 2 we used an open-ended question to ask whether children identify how the groups differed. This allowed for a broader and more informative range of responses. We reasoned that in Study 1, the questions we asked may have drawn children’s attention to differences in decision-making between the groups: An open-ended question would therefore test whether children spontaneously attend to the distinction between the two groups.

We also tested inferences about sharing in a different way: We asked children to predict how many resources a new member of each group (not featured in the stories) would share with another member of their group. The findings in Study 1 suggested that older children generalized from decision-making patterns to another attribute of the group—sharing. However, as mentioned above, it was unclear how the children interpreted this question since we did not specify what we meant by sharing. In Study 2 we directly investigate whether children expect more resource-sharing within egalitarian groups by asking children to predict the number of resources a new member of each group will share with another new member of their group. Both changes provided a stronger test of the hypothesis that children’s reasoning about the structure of social groups is theory-like, in that they spontaneously pick up on differences in decision-making patterns, and generalize behaviors across different contexts (i.e., decision-making to resource sharing).

As in Study 1, we asked children which group they would prefer to go camping with. In Study 2, children heard the same stories as in Study 1. Afterward, instead of asking, “*Which ones have someone in charge?*” we asked, “*What is different about the two groups?*” Instead of asking “*Which group shares more?*” we asked children to predict the number of resources that a new group member would share with another group member (i.e., “*Look, here is a new Wug, she has 5 strawberries, how many strawberries will she share with another Wug?*”). Study 2 was preregistered on the Open Science Framework (https://osf.io/puhn2). As in Study 1, we did not give children explicit information about the structure of the groups. We limited the study to older children because of the difficulty younger children had in Study 1 with tracking the structures of the two groups (see also the Supplemental Materials for a description of another study in which younger children failed to track the structure of the groups).

## METHOD

### Participants

We tested 32 children between the ages of 6 and 8 years (*M* = 7 years, 8 months; Min = 6 years and 2 weeks, Max = 8 years, 11 months). Thirteen parents said their child was a boy; 17 said their child was a girl. When asked to indicate racial background, 13 parents answered “White”; 1 answered “Asian”; 2 answered “Black”; 4 answered “White and Asian”; 12 did not answer the question. When asked about ethnicity, 3 answered Hispanic or Latino; 14 answered “Not Hispanic or Latino”; and 15 did not answer this question. Children were recruited from an existing lab database and resided mostly in the Boston metro area in the United States. We used a flexible stopping rule that we preregistered, which stated that we would stop once we achieved a Bayes Factor of at least 9 in our main analyses.

### Procedure

Study 2 was conducted over video chat. The description of the two groups (Wugs and Flurps) was the same as in Study 1. After children heard about the two groups, they answered two questions in a fixed order: “*What is different about the Wugs and the Flurps?*” and “*Did you notice anything else?*” If participants did not say something about decision-making in response to either question, we repeated the story once more and asked the questions once more. (Note, if a participant did not mention decision-making during the first round of questioning, it was coded as “other.”) Then we asked, “*Which group would you rather go camping with?*” and “*Why?*” Finally, children were presented with two new members of each group (the new characters had different hairstyles and hair colors from the characters presented in the camping stories). Five strawberries appeared next to one of the characters, and we asked the children, “*How many strawberries do you think this [Flurp/Wug] will share with another [Flurp/Wug]?*” The identity of the hierarchical group and whether children heard about the hierarchical group first were counterbalanced across participants.

### Coding

A research assistant who was unaware of the hypothesis of the experiment coded whether children’s responses mentioned decision-making when asked what was different about the two groups, and when the children were asked why they chose the group they chose. Some example responses that were coded as “decision-making” were: “*for yellow guys, everyone gets a turn choosing something; with the green guys, only the one with blue hair got to choose*”; “*Purple the Flurp* [referring to name of the Flurp with purple hair] *was the one who said everything, every one of the Wugs chose one thing*”; “*The Wugs cooperated together and each got a turn to pick something, for the Flurps, only Purple the Flurp got to decide.*” Some example responses that were not coded as decision-making included, “*The first thing that is different is that they have different colored bodies*”; “*The color.*” Children were also asked to give their reasons for why they preferred one group or the other, which was coded as either including decision-making or not. For example, “*because I would probably also get to choose something. They are kinder*”; “*they seem nicer because each of them got to choose not just one of them*” compared to “*it would pretty much be the same because mostly they are the same people except for their uniforms*.”

Text transcripts of the children’s answers can be found on the OSF page; see README file (https://osf.io/s6cu9/).

### Data Analysis

We used the program JASP (Love et al., 2019) to analyze the data in Study 2. We ran a two-sided Bayesian binomial test to investigate whether children answered the open-ended question by referring to decision-making structure more than half of the time and whether they chose the hierarchical or egalitarian group more than half of the time. We used a paired-sample Bayesian *t* test to ask whether children expected members of the egalitarian group to share more with one another than members of the hierarchical group. We did not expect to find an age effect in this study because we thought that children answering the question about the two groups may prompt even 6-year-olds to prefer the egalitarian group, so we did not preregister analyses that include age and we did not design a study to be powered to detect age effects. However, we include these analyses in the Supplemental Materials.

### Results and Discussion

#### What was different about the two groups?

**Figure 6.**
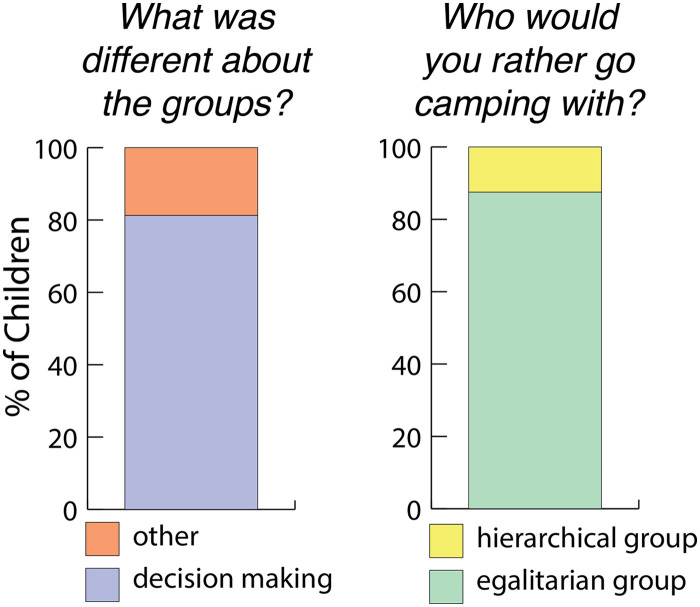
[left] Percentage of children who said something about the decision-making structure when asked what was different about the groups. [right] Percentage of children who chose the hierarchical/egalitarian group in Study 2.

When children were asked, “*What was different about the two groups?*” children were more likely to say that the groups differed in the way they made decisions compared to any other answer. Of the 32 children tested, 26 included decision-making in their answers (26/32, BF_10_ = 143.62 in favor of the alternative hypothesis that children said the two groups differed by decision-making more than half of the time; see Figure 6). Nineteen of these 26 children said decision-making the first time they were asked this question, five children said decision-making after the experimenter said, “*Did you notice anything else?*” and two of the children that included decision-making in their answers, did so after the experimenter repeated the story and asked the question again (one of these children heard the story twice because they answered the first two questions by saying, “I don’t know and one child heard the story twice because they failed to say anything about decision-making in their initial answers). Thirteen of the children also said that the groups differed because of their shirt colors, one said they didn’t differ, one child said that the difference was that one group came first, and one child said they differed because of the order they performed their tasks.

#### Which group would you rather go camping with?

Children chose the egalitarian group more often than the hierarchical group. Of the 32 children, 28 chose the egalitarian group (BF_10_ > 1,000; see Figure 6). In explaining why they would rather go camping with the egalitarian group, most of the children mentioned something about decision-making social structure (22/32, BF_10_ = 3.329). Of the 26 children who said that the two groups differed because of decision-making, 25 of them chose the egalitarian group (BF_10_ = 18539.99; note this last analysis was not preregistered).

#### How many resources will the characters share with a fellow group member?

**Figure 7.**
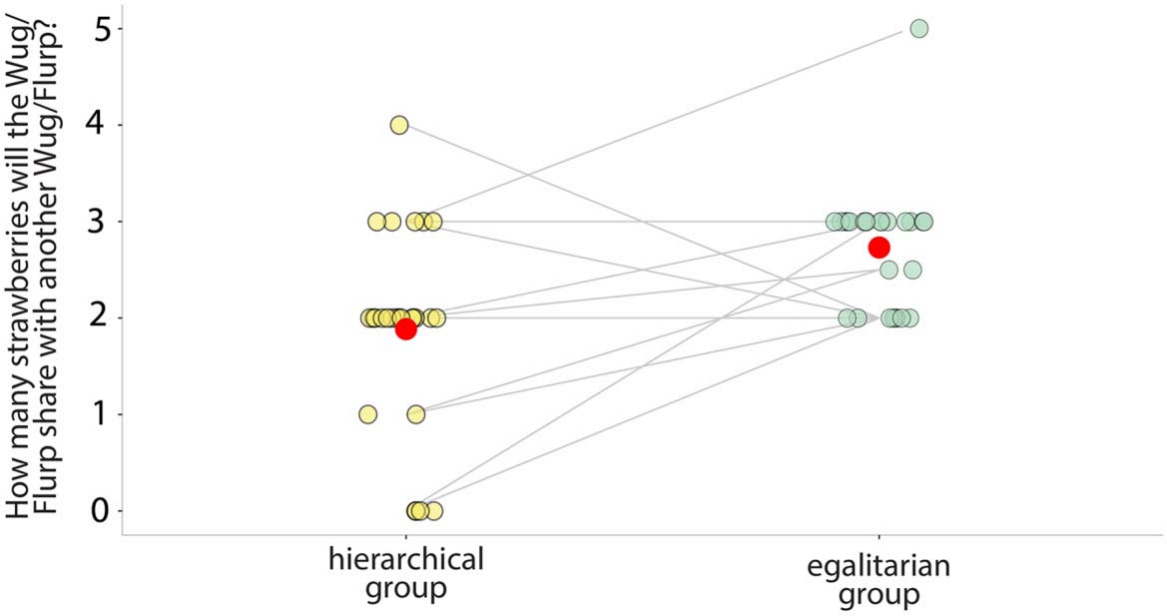
**Answers to the sharing question in Study 2.** Dots are individual participant answers (between 0 and 5), and lines connect answers from a single child. The red dots are the mean.

When asked to guess how many resources a member of each group would share with another member of their group, children consistently said that the egalitarian group would share more resources. Among our preregistered analyses was one that only included children who said that the two groups differed by decision-making structure. For these 26 children, we found strong evidence that they thought the member of the egalitarian group would share more resources than the member of the hierarchical group (*M*_hierarchical_ = 1.885, *SD* = 1.033; *M*_egalitarian_ = 2.731, *SD* = 0.652, BF_10_ = 21.132; see Figure 7). When we include all 32 children in the analysis (this was not preregistered), we still find moderate evidence that children said that the member of the egalitarian group would share more than the member of the hierarchical group (*M*_hierarchical_ = 1.984, *SD* = 1.004; *M*_egalitarian_ = 2.656, *SD* = 0.641, BF_10_ = 8.992).

## GENERAL DISCUSSION

In these studies, 6- to 8-year-old children, but not 4- and 5-year-old children, distinguished groups based on decision-making patterns, distinguishing between hierarchically organized groups, where one character made all the decisions, and nonhierarchically organized groups, where different decisions were made by different characters. The older children also inferred that members of the egalitarian group would share more resources with one another than members of the hierarchical group. Moreover, 6- to 8-year-old children preferred to interact with the group that shared decision-making. Thus, children’s early intuitive sociology goes beyond identifying group membership, and beyond reasoning about roles within dyadic relationships, to include knowledge about how groups are structured.

Why did 4- and 5-year-old children fail to distinguish between the two groups? The data may reflect a genuine representational change, such that younger children lack knowledge that groups can be structured in different ways. However, the task was also difficult: it required children to track several actions and individuals. Thus, it is perhaps not surprising that younger children had difficulty tracking the two groups in Study 1 (and see the Supplemental Materials, Study S1). Similar age-related changes have been found in other studies. For example, when asked to explain success in a rigged game, younger children tend to give person-related explanations (e.g., “he is strong”) while older children give situational explanations (e.g., “the game is rigged”; Peretz-Lange et al., 2021). There may be a shift in children’s reasoning such that only older children consider external influences, such as group structure or rigged games when predicting people’s behavior. It is also possible that domain-general development such as working memory helped older children in the current studies, but also helped them learn about more complicated aspects of their social environment, such as group structure. Thus, these two ways of interpreting the data may be difficult to disambiguate.

Where do the intuitions of older children come from? It is unlikely that the older children have been explicitly taught that groups who have leaders share less with one another. Children may have learned through experience that these two attributes go together: it is possible that groups with leaders do share less. However, there are many hierarchical groups in which the opposite is true (e.g., in a nuclear family setting). It is possible that when children learn about a group that shares in decision-making, they imagine that people in that group feel closer to one another or more cooperative, and thus any two individuals would be more likely to share more with one another. Likewise, children may imagine a hierarchical group to be made up of competitive relationships. Or it could be that members of a group who share in decision-making are more likely to have equal status, and thus distribute resources in a more even way. A related possibility is that children infer a third attribute that causes the group to be structured hierarchically and causes group members to be less generous. Children may assume that the characters in the egalitarian group were more trusting of one another, and thus more generous. Adults, sampled from a similar population as the children we tested here, believe that resource scarcity leads both to immoral behavior and authoritarian social arrangements (Nettle & Saxe, 2021), thus children may assume that authoritarian social arrangements (i.e., the hierarchical decision-making arrangement) lead to immoral behavior including less sharing.

Why did the older children in our studies prefer nonhierarchical groups? Perhaps children assumed that they would not be able to make decisions in the hierarchical group and preferred to be in situations where they could make decisions. Moreover, adults often encourage children to take turns, modeling a cultural ideal of egalitarianism. However, children also find themselves in many situations where decision-making power is concentrated: teachers make decisions for classrooms, parents make decisions for families, and older children may make decisions for younger ones in mixed-age playgroups. Future research could directly test whether children’s preferences for different types of group structures relate to explicit teaching (e.g., being told not to be “bossy”), or experience (e.g., being in a classroom where students get to make more decisions). Moreover, children’s preferences may vary depending on the context, as adults’ preferences seem to do (e.g., Nettle & Saxe, 2020). For example, in pedagogical settings, children may prefer hierarchical structures because they want to learn from the most knowledgeable people. Children might also prefer hierarchical groups in group competitions where having a leader could make group coordination faster. Recent work shows that when reasoning about hypothetical societies, contexts such as war or scarcity affect adults’ preferences for the distribution of resources (Nettle & Saxe, 2020). However, less is known about the contexts in which adults or children prefer different distributions of power.

Future research could also investigate children’s evaluations of hierarchy depending on the avenues that lead to differences in power. For example, when do children differentiate hierarchical groups in which the leader is elected from those in which one assumes power without consensus?

Future research could also investigate whether children are aware of the structure of their social groups. As children get older, they have more control over how their groups are structured. In adults, ingroup bias correlates strongly with a preference for hierarchical social structures (Pratto et al., 1994). Understanding how preferences for egalitarian or hierarchical structures develop in childhood may lead to a better understanding of ingroup bias and its attendant social problems of prejudice and discrimination (Beelmann & Heinemann, 2014; Skinner & Meltzoff, 2019). For example, when adult workers are randomly assigned to participate in groups that are worker led (as opposed to supervisor led), it not only affects how productive they are at their job but also influences attitudes such as “belief in a just world” and their participation in politics outside of their workplace (Wu & Paluck, 2020, 2022). Future work could investigate whether children who participate in groups with varying group structures show related effects.

One limitation of this study is that we only tested children in two geographical areas within the United States—Orange County and the Boston Metro Area. Ideas about authority, status, and forms of social organization vary widely across cultures. Even within cultures, ideas about authority vary with factors like religious background (Bulbulia et al., 2013) and political affiliation (Graham et al., 2009). An important question for future studies is whether children’s preferences for egalitarian or hierarchical groups covary with the same demographics that predict adult attitudes (Terrizzi, 2020).

While these studies raise many questions, they establish that by age 6, children are sensitive to group structure. Children paid attention to the frequency with which different individuals made decisions, and they compared groups on this basis. They preferred shared decision-making. Importantly, the children in these studies also inferred that characters in an egalitarian group would be more generous, showing that their beliefs about group structure are theory-like in that they made inferences from one domain (decision-making) to another (resource distribution). This study is a first step in understanding a broad set of abilities to abstractly reason about social structures.

## ACKNOWLEDGMENTS

Thank you to the research assistants including Judith Gallardo, Anne Brunson, Eden Howard, Silvia Yamilee Navarro Hernandez, Pontea Doroudian, Hasmik Mehrabyan, Heaven Howse, Ghadeer Alabbas, and Mikayla Hand. Thank you to the Cambridge Writing Workshop who gave excellent feedback on this manuscript (Rhea Howard, Brandon Woo, Leyla Tarhan, Lauren DiNicola, Shari Lui, and Maya Rosen). Thank you to the Discovery Cube of Orange County.

## FUNDING INFORMATION

AJT, Eunice Kennedy Shriver National Institute of Child Health and Human Development (https://dx.doi.org/10.13039/100009633), Award ID: 1F32HD096829. Study 3 is based upon work supported by the Center for Brains, Minds and Machines, funded by the National Science Foundation STC Award CCF-1231216 and Siegel Foundation Award S4881.

## AUTHOR CONTRIBUTIONS

AJT: Conceptualization, Experimental design, Writing, Project Management, Data Analysis; VM: Experimental Design, Writing; ES: Project Management, Writing; BFT: Experimental Design, Writing; PKP: conceptualization; experimental design, writing; BWS: Experimental design, Writing, Project Management, Data Analysis.

## Supplemental Materials

https://osf.io/puhn2

https://osf.io/s6cu9/
