# Supplementary material for: Intuitive Sociology: Children Recognize Decision-Making Structures and Prefer Groups With Less-Concentrated Power
Source: Open Mind (Camb). 2022 Jul 1;6:25–40. doi: 10.1162/opmi_a_00053 (PMC9692051; doi:10.1162/opmi_a_00053)
Supplement: Supplementary file 1 [file opmi-06-25-s001.pdf]

Children recognize decision-making structures and prefer groups with less concentrated power

Supplemental Materials and Analyses

Ashley Thomas, Vivian Mitchell, Emily Sumner, Brandon Terrizzi, Paul Piff, Barbara Sarnecka

12/15/2021

Contents

1 Extra Analyses, Plots, and Results for Study 1 **3**

1.1 Model . . . 3

1.2 Results . . . 3

1.2.1 *Which ones had someone charge?′* . . . 3

1.2.1.1 ROPE and PD analysis for *Which ones had someone in charge?′* . . . 4

1.2.1.2 Age differences for *Which ones had someone in charge?* . . . 6

1.2.2 *Who Shares more?′* . . . 7

1.2.2.1 Rope and PD analsysis for *Who share more?′* . . . 7

1.2.2.2 Age differences in *Who Shares More?′* . . . 9

1.2.2.3 Order differences for *Who shares more?′* . . . 10

1.2.3 *Would you rather be a Wug or a Flurp′* . . . 10

1.2.3.1 ROPE and PD analysis for *Would you rather be a Wug or a Flurp?′* . . 10

1.2.3.2 Age differences for *Would you rather be a Wug or a Flurp?′* . . . 12

1.2.4 *Who would you rather go camping with?* . . . 13

1.2.4.1 ROPE and PD analysis for *Who would your rather go camping with?′* . 13

1.2.4.2 Age differences for *Who would you rather go camping with?′* . . . 15

1.2.4.3 Order differences for *Who would you rather go camping with?′* . . . 15

1.2.4.4 Gender differences for *Who would you rather go camping with?′* . . . 15

1.2.5 *Who was in Charge?′* . . . 16

1.2.5.1 ROPE and pd analyses for *Who was in Charge?′* . . . 16

1.2.5.2 Age differences for *Who was in Charge?* . . . 18

1.3 Extra Analyses for Study 1 . . . 19
1.3.1 *Would you rather be a Wug or a Flurp?* . . . 19
1.3.1.1 ROPE and pd analyses for *Would you rather be a Wug or a Flurp?* . . . 19
1.3.1.2 Breaking down by Factor for *Would you rather be a Wug or a Flurp?* . 21
1.3.2 *Who would you rather go camping with?* . . . 22
1.3.2.1 ROPE and pd analyses for *Who would you rather go camping with?* . . 22
1.3.2.2 Breaking down by Factor for *Who would you rather go camping with?* 24

2 Extra Analyses, Plots, and Results for Study 2 **25**
2.1 Results . . . 25
2.1.1 *Who would you rather go camping with?′* . . . 25
2.1.1.1 ROPE and PD analysis for *Who would you rather go camping with?′* . 25
2.1.1.2 Age differences for *Who would you rather go camping with?′* . . . 27
2.1.1.3 Explanation differences for *Who would you rather go camping with?′* . 28
2.1.2 *What was different about the two groups?′* . . . 28
2.1.2.1 ROPE and PD analysis for *What was different about the two groups?′* . 28
2.1.2.2 Age differences for *What was different about the two groups?′* . . . 30
2.1.2.3 Order differences for *What was different about the two groups?′* . . . 31
2.1.2.4 Gender differences for *What was different about the two groups?′* . . . 31

3 Supplemental Study 1 (Study S1) **31**
3.1 Methods . . . 32
3.1.1 Participants . . . 32
3.1.2 Procedure . . . 32
3.1.3 Results and Discussion . . . 32
3.1.4 *Which ones had someone in Charge?* . . . 32
3.1.4.1 ROPE and PD analysis for *Which ones had someone in charge?′* . . . 32
3.1.4.2 Age differences for *Which ones had Somoene in Charge?* . . . 34
3.1.5 *Who would you rather go camping with?* . . . 35
3.1.5.1 ROPE and PD analysis for *Who would your rather go camping with?′* . 35
3.1.5.2 Age differences for *Who would you rather go camping with?′* . . . 37
3.1.6 *Would you rather be a Wug or a Flurp′* . . . 38
3.1.6.1 ROPE and PD analysis for *Would you rather be a Wug or a Flurp?′* . . 38
3.1.6.2 Age differences for *Would you rather be a Wug or a Flurp?′* . . . 39

3.1.7 *Who Shares more?'* . . . 40
3.1.7.1 Rope and PD analsysis for *Who share more?'* . . . 40
3.1.7.2 Age differences in *Who Shares More?'* . . . 42
3.1.7.3 Order differences for *Who shares more?'* . . . 43
3.1.8 *Who was in Charge?* . . . 43
3.1.8.1 ROPE and pd analyses for *Who was in Charge?* . . . 43
3.1.8.2 Age differences for *Who was in Charge?* . . . 45
3.1.8.3 Order differences in *Who was in Charge?'* . . . 46
3.2 Discussion . . . 46

See OSF page for data dictionary, code for this document, data sets, stimuli etc. https://osf.io/s6cu9/

1 Extra Analyses, Plots, and Results for Study 1

1.1 Model

More discussion about the analysis approach can be found in the Main text.

The general format of our models were:: | **MODEL** <- brm (**Outcome_Variable**~Age+Gender+Order_1, data = Study1, family | = bernoulli (link=logit), iter= 5000, warmup= 1000, thin= 1,chains=6,save_pars = save_pars(all = TRUE))

For each of the dependent measure questions, we first fit the model, then show the posterior predictive distribution which can be used to judge whether the model is a good fit. Then we plot the posteriors and overlay the ROPE (region of practical equivalence). For each model we will also show the percentage of the posterior that falls in this region. These percentages are reported in the Main Text. Then we plot and calculate the 'probability of direction' which tells you how probable it is that each factor is either positive or negative. Then, if we have evidence that any of the factors had an affect we use the proportionBF function to perform a Bayesian binomial test. Unless otherwise stated, the null hypothesis is 50%. (See Main Text for more details)

1.2 Results

1.2.1 *Which ones had someone charge?'*

First we'll fit a model and check to see if the model is a 'good fit' using a posterior predictive distribution (pp_check).

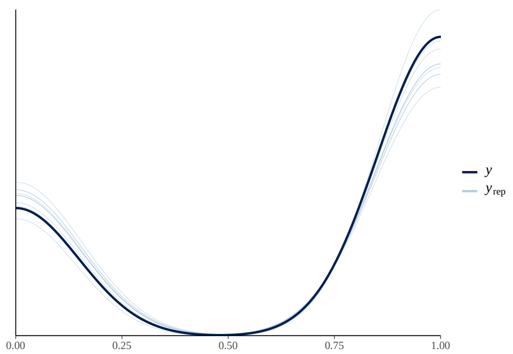

This looks pretty good (the light blue lines are not deviating much from dark blue line)

1.2.1.1 ROPE and PD analysis for *Which ones had someone in charge?'* _

Now we will plot the posteriors, and overlay the ROPE (region of practical equivalence). Note that the ROPE was pre-registered, but it is subjective. This analysis was done after reviews, so we had already looked at and analyzed the data. We chose this approach after realizing the type of model we had fit was not appropriate for binomial data. Here the ROPE is -.01 to .01, which is a null region. In these models, Order is whether children heard about the hierarchy group first or second; Age is in years, and Gender is whether their parents reported their child's gender as 'girl' or 'boy'. If they chose the hierarchical group, their answers were coded as '1', If they chose the egalitarian group, their answers were coded as '0' unless otherwise stated. This means that in the posterior plots, positive numbers (to the right) mean they were more likely to choose the hierarchical group, lower numbers (to the left) mean they were more likely to choose the egalitarian group, unless otherwise indicated.

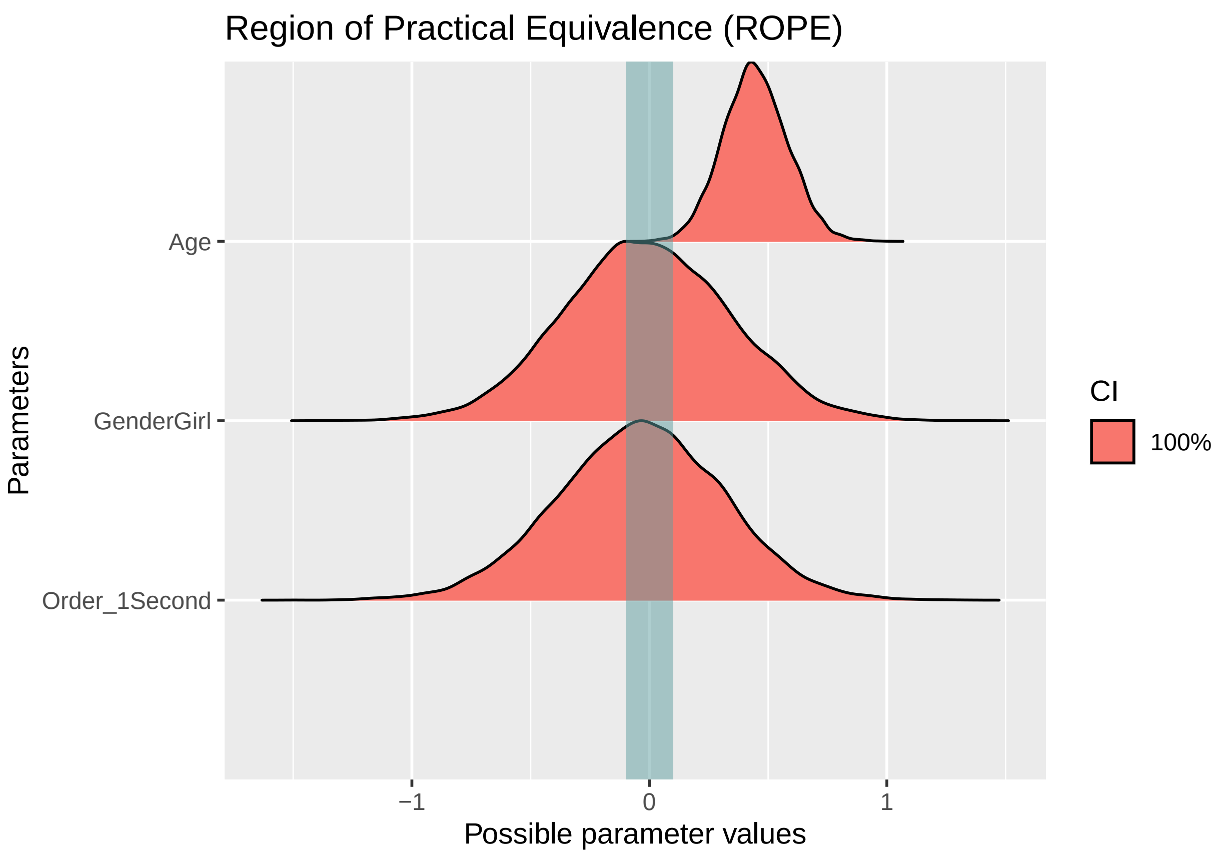

Based on these plots, it looks like it is is probable that Age had an effect, such that older children were more likely to choose the group with a leader (the posterior is shifted to the right of the null region). It is plausible that Order and Gender did not have an effect, since the peak of the posterior distributions lie within the null regions. However, this does not mean that we have strong evidence since most of the posterior lies outside of the null region. Next we'll calculate the percent of the posterior that falls within this region.

ROPE Percentages

Which ones had someone in charge?

Parameter	ROPE_Percentage
b_Intercept	1.19375
b_Age	0.39375
b_GenderGirl	22.05625
b_Order_1Second	23.02500

A very small percentage of 'Age' posteriors fall into this region (0.39%) so it is probable that Age had an effect, such that older children were more likely to choose the hierarchical group.

Next we calculate and plot a probability direction score, using the posteriors.

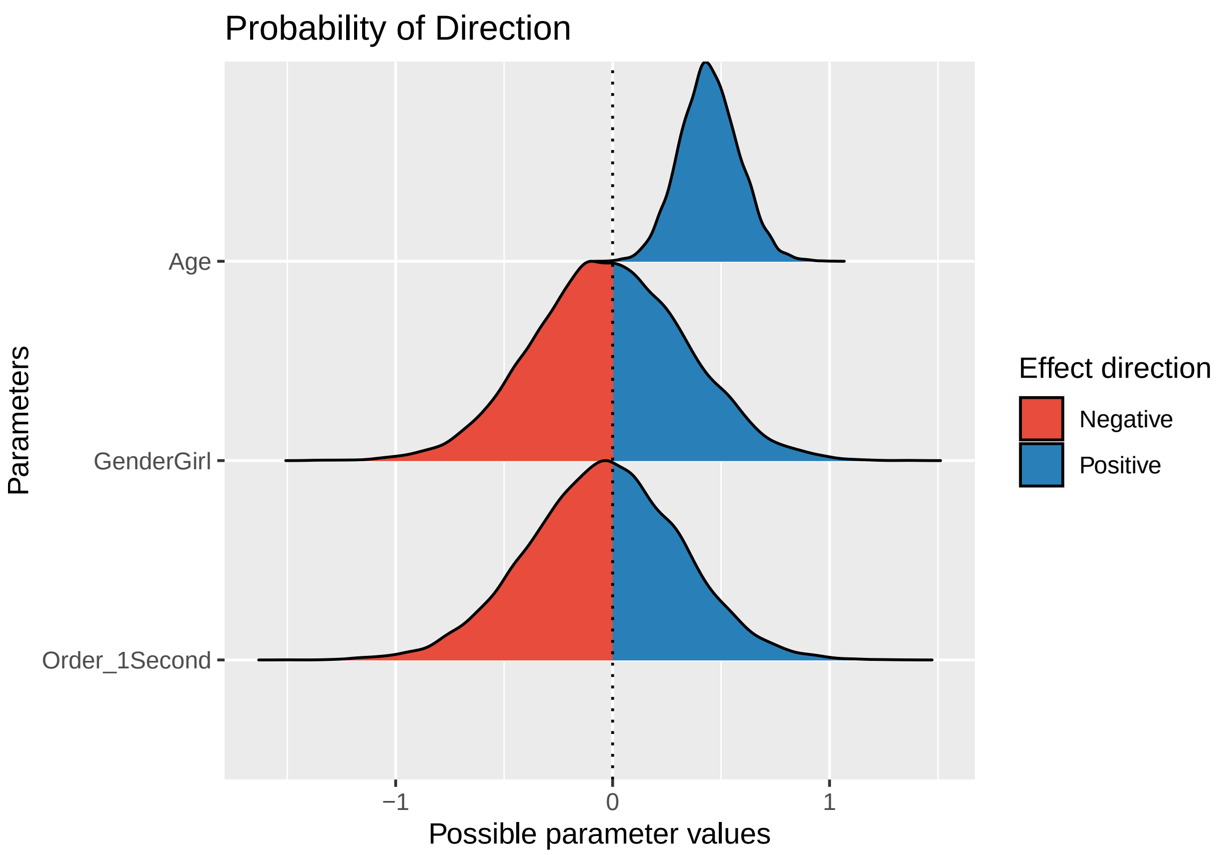

PD Percentages

Which ones had someone in charge?

Parameter	pd
b_Intercept	98.09375
b_Age	99.98125
b_GenderGirl	51.70000
b_Order_1Second	54.45000

The probability of the direction of the effect is close to 100% for age (meaning it is very likely that older children chose the hierarchical group), but 50/50 for Order and Gender. Thus, this second analysis agrees with the ROPE analysis. Age likely affected children's answers, but not Order and Gender

1.2.1.2 Age differences for *Which ones had someone in charge?* _

Since Age had an effect, we'll look at how each age group answered this question, separating the children by year.

Age Differences

Which ones had Someone In Charge?

Age	chose_hier	chose_egal	total	%_Chose_Hier	Bayes_Factors
4	27	15	42	64.28571	1.6064473
5	16	20	36	44.44444	0.4602212
6	27	8	35	77.14286	38.0143146
7	26	5	31	83.87097	249.5636434
8	23	3	26	88.46154	440.9453388

The Bayes Factors here are calculated using proportionBF in the BayesFactor package (see Main text). Here, we have strong evidence that 6- to 8-year-olds chose the group with the leader, and inconclusive evidence as to whether 4 and 5 year olds were at chance.

1.2.2 *Who Shares more?'*

Again, fit model and check to see if the model is a 'good fit' using a posterior predictive distribution (pp_check)

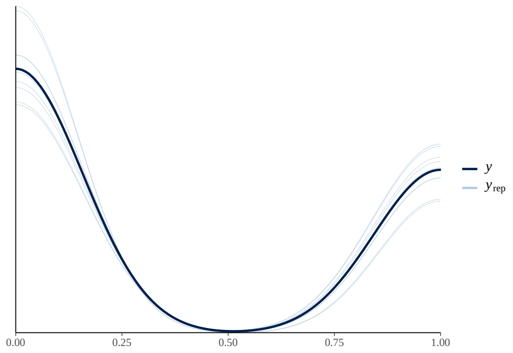

Looks pretty good (light blue lines are not deviating much from dark blue line)

1.2.2.1 Rope and PD analsysis for *Who share more?'*

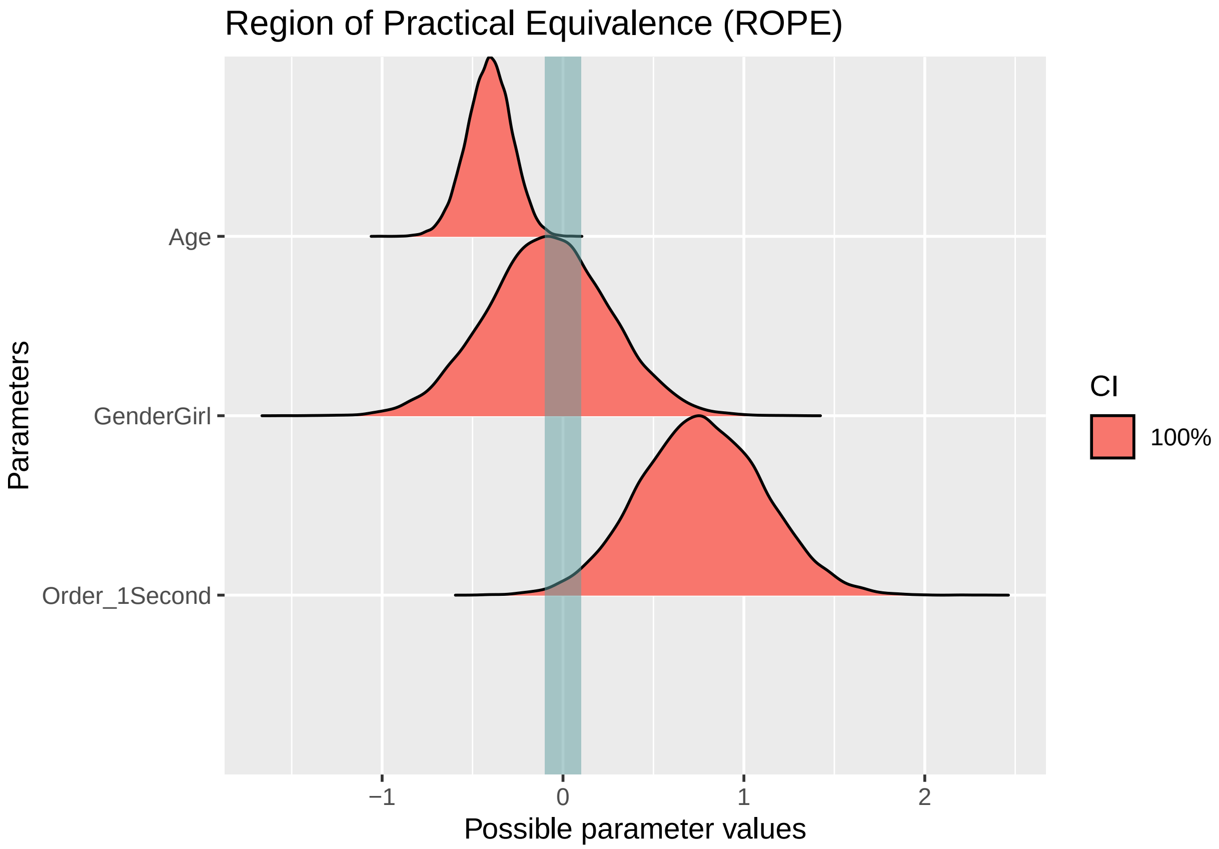

Age seems to have an affect on children's answers, Order may have had an effect such that children who heard about the hierarchical group first were more likely to choose the egalitarian group when asked "Who shares more?"

ROPE Percentages

Who Shares More?

Parameter	ROPE_Percentage
b_Intercept	1.60625
b_Age	0.52500
b_GenderGirl	23.00000
b_Order_1Second	1.83750

A very small percentage of 'Age' posteriors fall into this region so it is likely that Age has an affect. Likewise a small percentage of the 'Order'falls into the region.

Now we can calculate and plot a probability direction score, using the posteriors

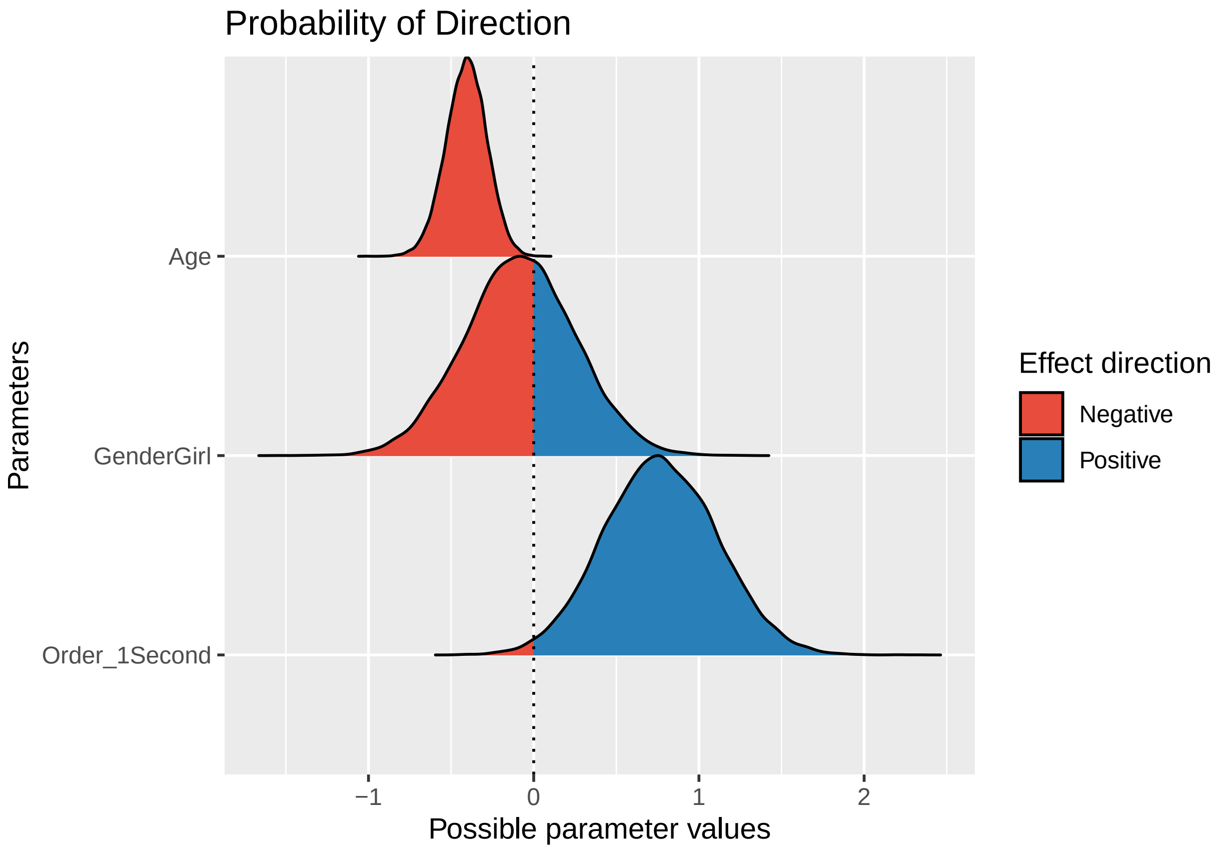

PD Percentages

Who shares more?

Parameter	pd
b_Intercept	97.69375
b_Age	99.97500
b_GenderGirl	59.81875
b_Order_1Second	98.93125

Here the probability of the direction is close to 100% for age and Order, but closer to 50% for Gender.

1.2.2.2 Age differences in *Who Shares More?'*

Age Differences

Who Shares More?

Age	chose_hier	chose_egal	total	%_Chose_Hier	Bayes_Factors
4	23	19	42	54.76190	0.4211080
5	17	19	36	47.22222	0.3995077
6	10	25	35	28.57143	6.3464925
7	10	21	31	32.25806	2.1124592

8	5	21	26	19.23077	29.9647787

We have strong evidence that 8 year-olds chose the group without a leader more often for this question, weak evidence that 7 year-olds did so and moderate evidence that 6 year-olds did so.

1.2.2.3 Order differences for *Who shares more?'*

Order Differences

Who Shares More?

Order_1	chose_egal	chose_hier	total	%_Chose_Hier	Bayes_Factors
First	55	23	78	29.48718	137.9479872
Second	50	42	92	45.65217	0.3477838

Children who heard about the hierarchical group first, were much more likley to choose the egalitarian group when asked who shares more.

1.2.3 *Would you rather be a Wug or a Flurp'*

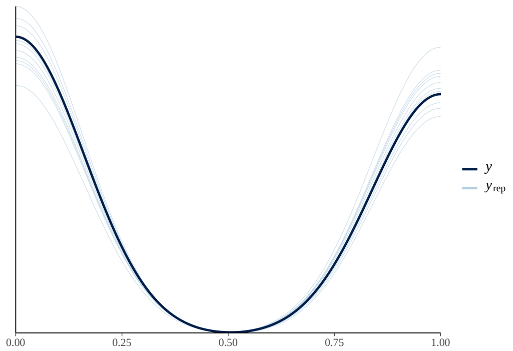

Looks pretty good (light blue lines are not deviating much from dark blue line)

1.2.3.1 ROPE and PD analysis for *Would you rather be a Wug or a Flurp?'*

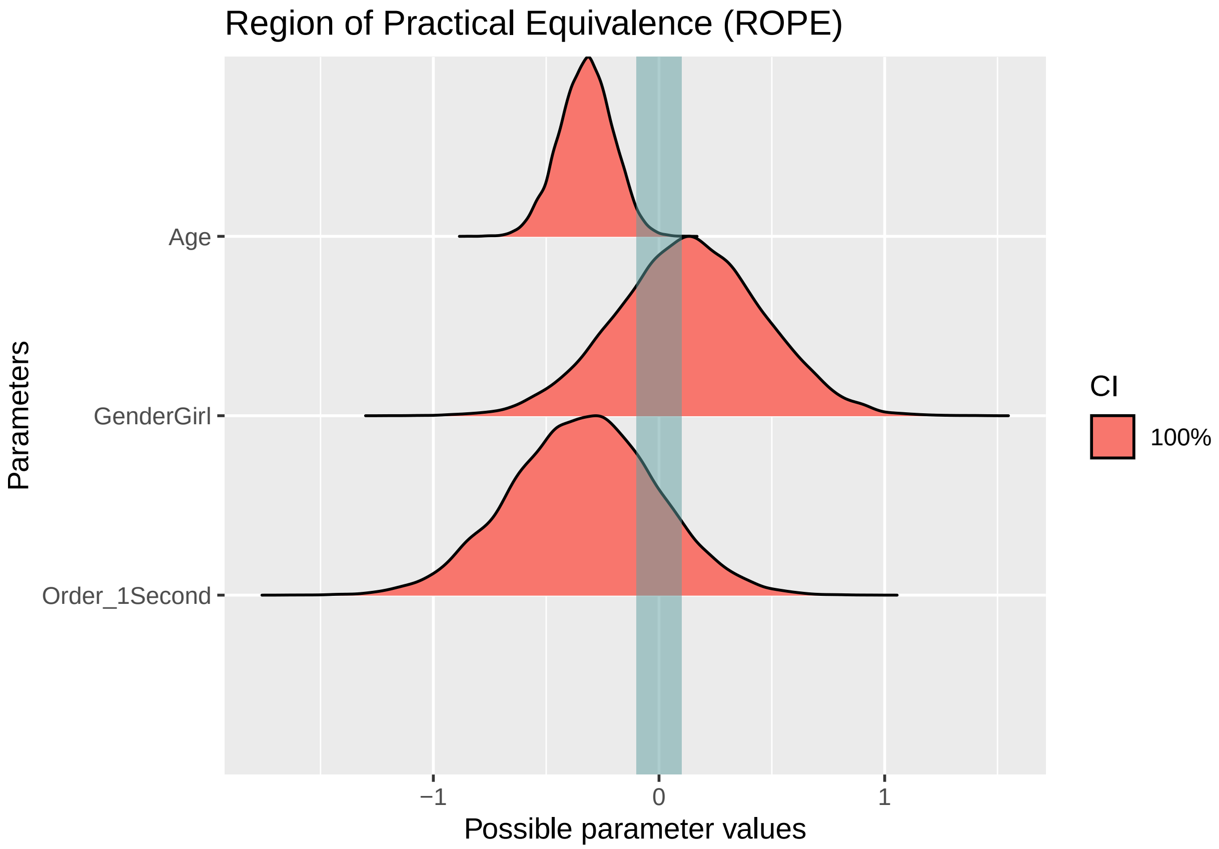

Age likely affected children's answers, but we have inconclusive evidnece as to whether Gender or Order affected children's answers.

ROPE Percentages

Would you rather be a Wug or a Flurp?

Parameter	ROPE_Percentage
b_Intercept	0.57500
b_Age	2.46250
b_GenderGirl	22.35000
b_Order_1Second	14.60625

A very small percentage of 'Age' posteriors fall into this region so it is likely that Age has an affect.

Now we can calculate and plot a probability direction score, using the posteriors

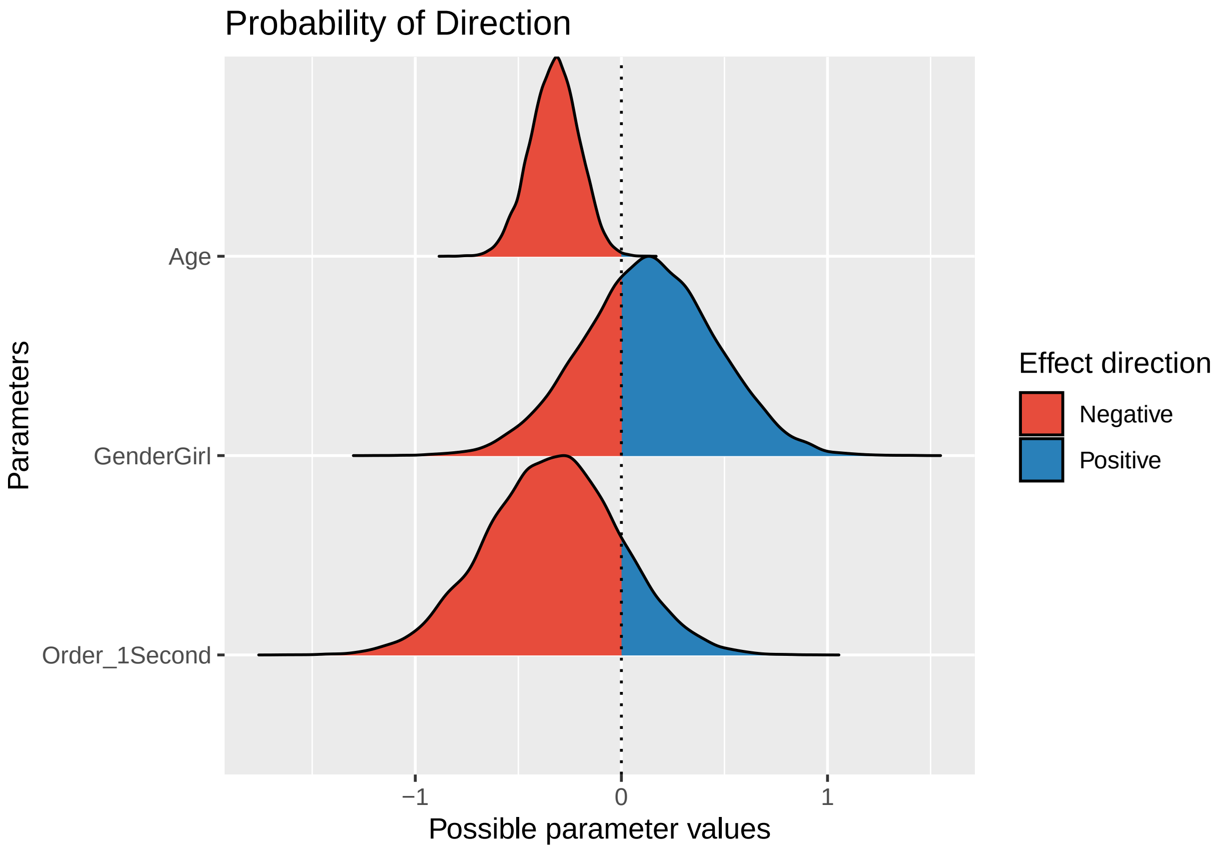

PD Percentages

Would you rather be a Wug or a Flurp?

Parameter	pd
b_Intercept	99.3625
b_Age	99.8000
b_GenderGirl	66.6125
b_Order_1Second	85.0000

Here the probability of the direction is close to 100% for age, but closer to 50% Order and Gender.

1.2.3.2 Age differences for *Would you rather be a Wug or a Flurp?'*

Age Differences

Would you rather be a Wug or a Flurp?

Age	chose_hier	chose_egal	total	%_Chose_Hier	Bayes_Factors
4	21	21	42	50.00000	0.3570721
5	22	14	36	61.11111	0.8141986
6	17	18	35	48.57143	0.3903255
7	9	22	31	29.03226	4.1524556

8	7	19	26	26.92308	4.3678216

Thus we found moderate evidence that 7 and 8 year olds chose the egalitarian group more often than the group with a leader when asked whether they would like to be a wug or a flurp

1.2.4 *Who would you rather go camping with?*

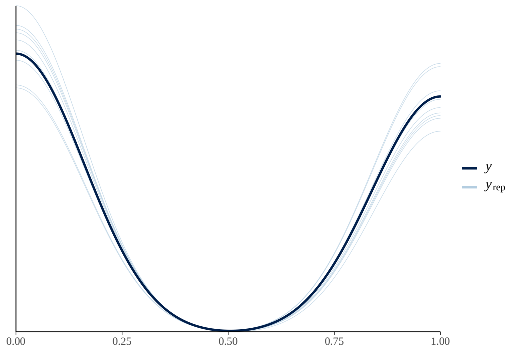

Looks pretty good (light blue lines are not deviating much from dark blue line)

1.2.4.1 ROPE and PD analysis for *Who would your rather go camping with?'*

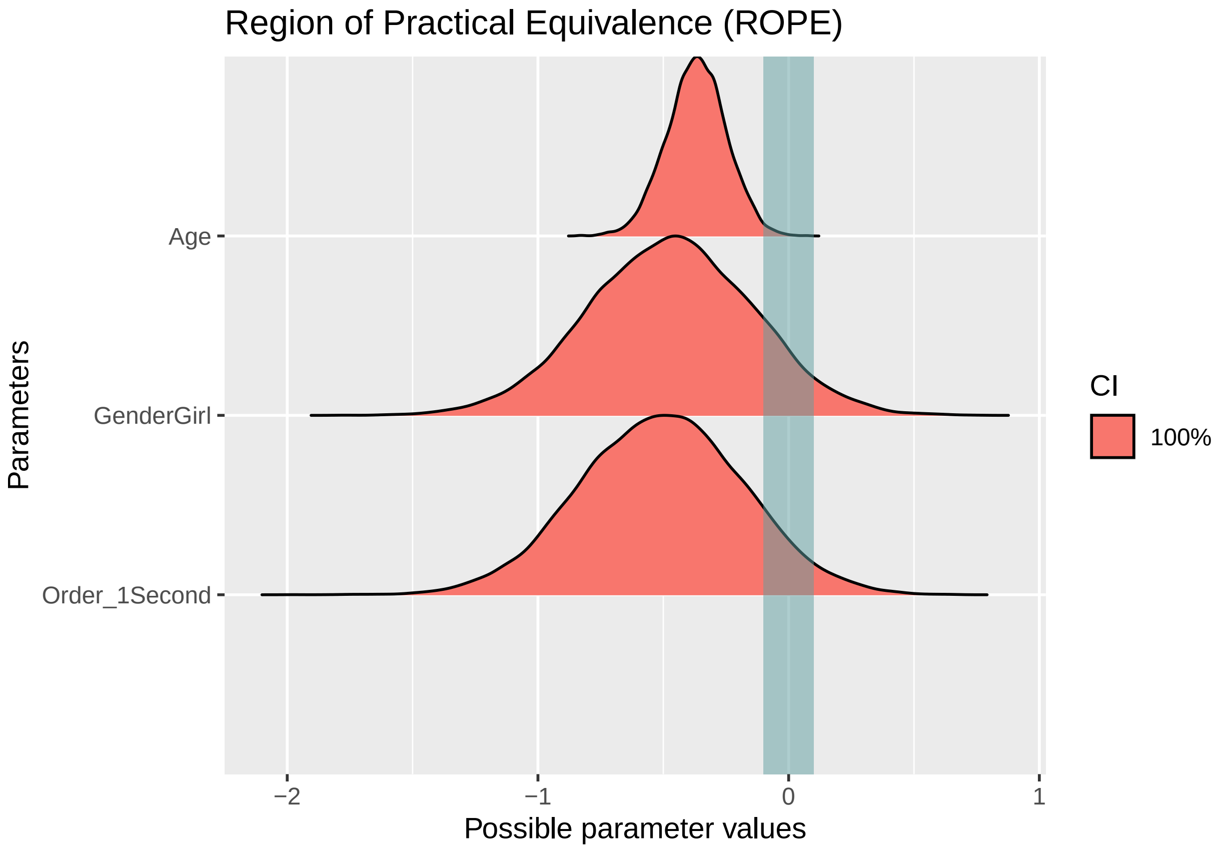

These plots suggest that older children were more likely to choose the egalitarian group than younger children. The posteriors for Gender and Order are also shifted to the left, suggesting that girls were potentially less likely than boys to choose the group with a leader, and that hearing about the hierarchical group second maybe made it so children were more likely to choose the egalitarian group. But this does not provide strong evidence since most of the posterior lies on either side of the ROPE.

ROPE Percentages

Who would your rather go camping with?

Parameter	ROPE_Percentage
b_Intercept	0.05000
b_Age	1.07500
b_GenderGirl	9.04375
b_Order_1Second	7.54375

A very small percentage of 'Age' posteriors fall into this region so it is likely that Age has an affect. The percentage of the Order and Gender posteriors that lie outside of the ROPE is also small, but quite a bit over the pre-registered cut-off so we'll take this as inconclusive evidence.

Now we can calculate and plot a probability direction score, using the posteriors

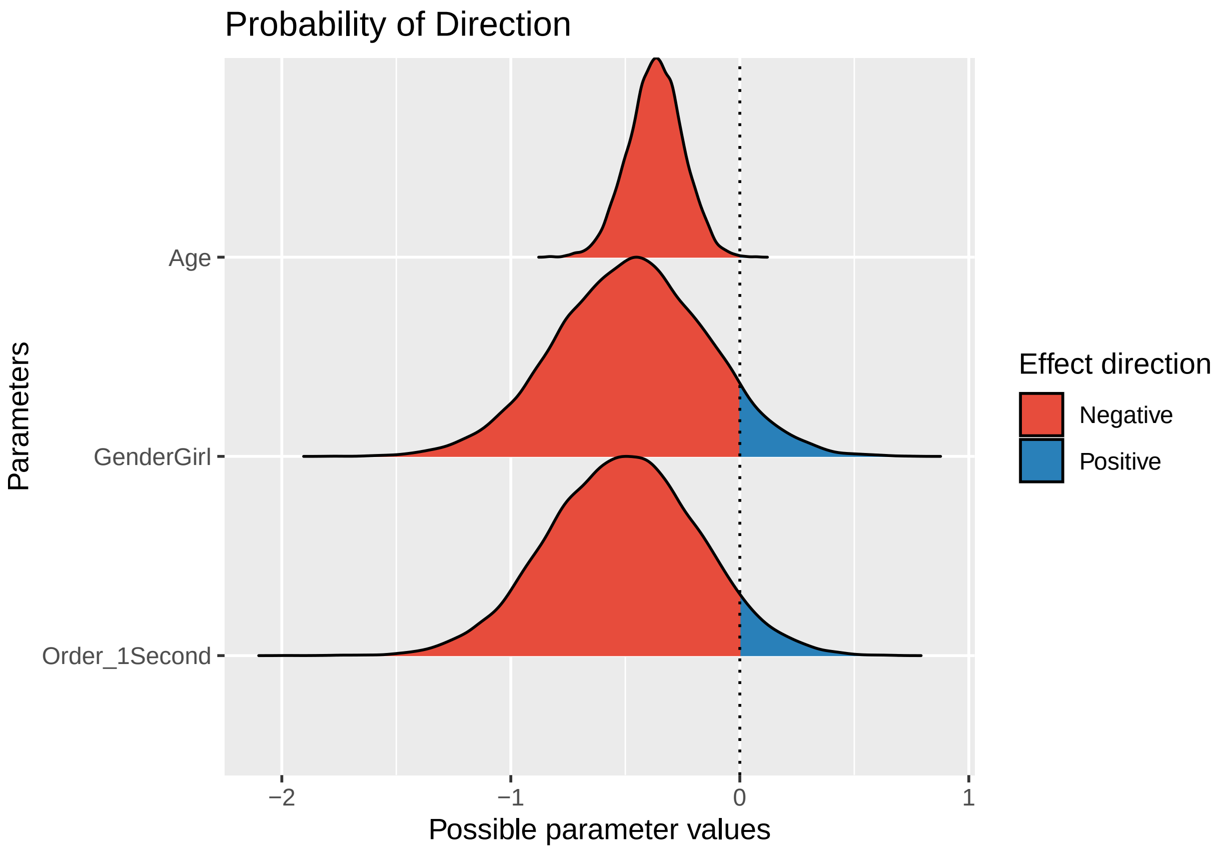

PD Percentages

Who would you rather go camping with?

Parameter	pd
b_Intercept	99.97500
b_Age	99.90625
b_GenderGirl	92.73750
b_Order_1Second	94.21250

The probability of the direction is close to 100% for age, and the probability of direction is also high for Order and Gender.

1.2.4.2 Age differences for *Who would you rather go camping with?'*

Since we found an Age effect, we'll look at differences between the age groups (also reported in the MS)

Age Differences

Who Would You Rather Go Camping With?

Age	chose_hier	chose_egal	total	%_Chose_Hier	Bayes_Factors
4	28	14	42	66.66667	2.7957147
5	14	22	36	38.88889	0.8141986
6	17	18	35	48.57143	0.3903255
7	13	18	31	41.93548	0.5668421
8	6	20	26	23.07692	10.5120228

We have strong evidence that 8 year-olds chose the egalitarian group, very weak evidence that 4 year olds chose the hierarchical group, and inconclusive evidence for the rest.

1.2.4.3 Order differences for *Who would you rather go camping with?'*

Since we had inconclusive evidence for 'Order' we'll look at how children answered depending on the order they heard the stories.

Order Differences

Who Would You Rather Go Camping With?

Order_1	chose_hier	chose_egal	total	%_Chose_Hier	Bayes_Factors
First	41	37	78	52.56410	0.2981668
Second	37	55	92	40.21739	1.3108354

Children who heard about the hierarchical group first, were slightly less likely to choose the hierarchical group. This may reflect a Recency bias.

1.2.4.4 Gender differences for *Who would you rather go camping with?'*

Since we had inconclusive evidence for 'Gender' we'll look at how children of different genders answered the question.

Gender Differences

Who Would You Rather Go Camping With?

Gender	chose_hier	chose_egal	total	%_Chose_Hier	Bayes_Factors
Boy	43	39	82	52.43902	0.2901663
Girl	35	53	88	39.77273	1.4364604

Girls may have been more likely to choose the egalitarian group than boys.

1.2.5 *Who was in Charge?'*

This analysis was not included in the main script. We also asked children to tell us which character was in charge.

Again, fit model and check to see if the model is a 'good fit' using a posterior predictive distribution (pp_check)

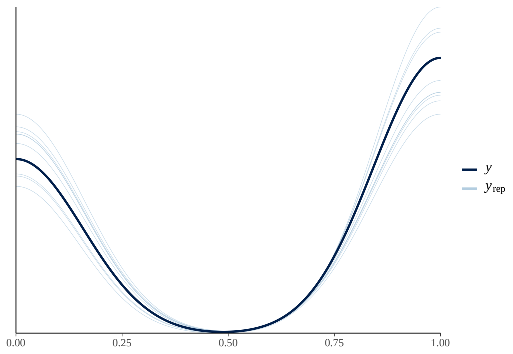

Looks pretty good (light blue lines are not deviating much from dark blue line)

1.2.5.1 ROPE and pd analyses for *Who was in Charge?'*

Now we will plot the posteriors with the ROPE (region of practical equivalence). Note the ROPE was decided ahead of time, but it is somewhat arbitrary.

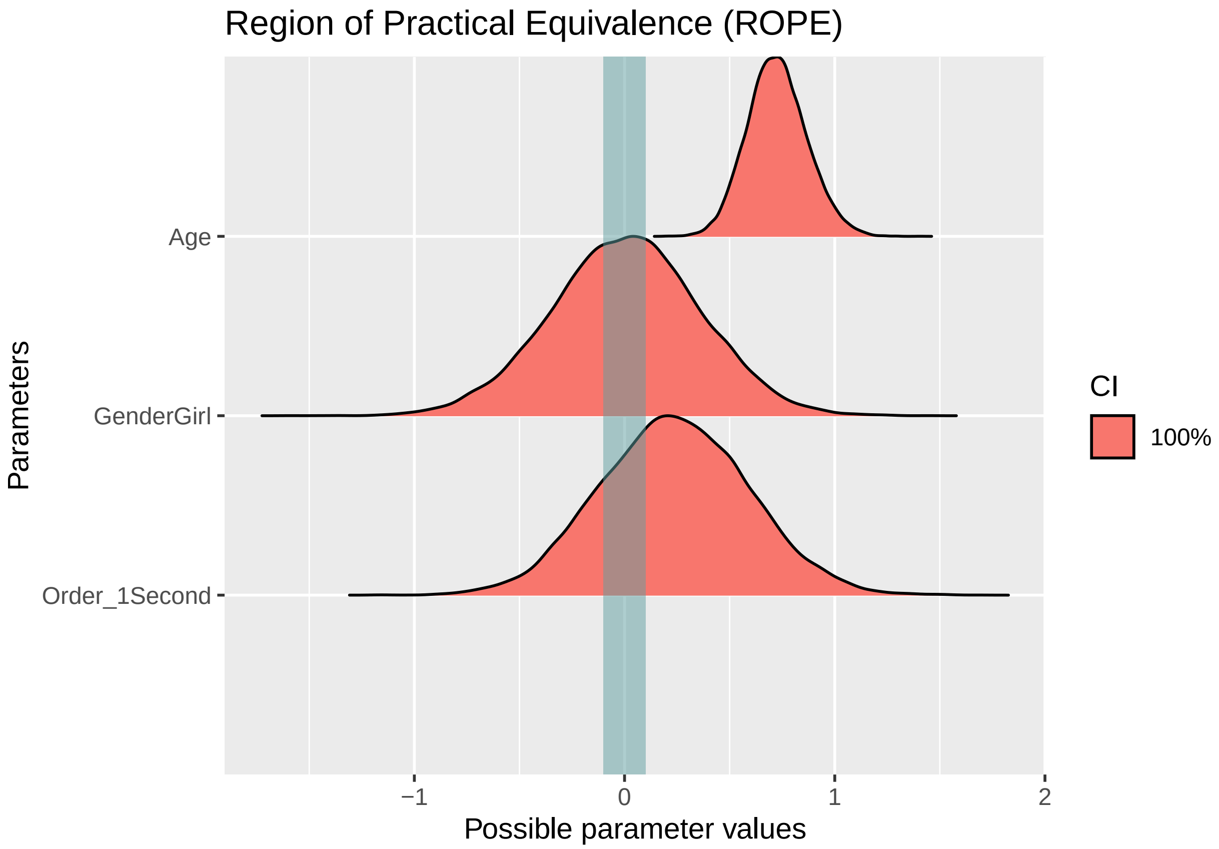

Age seems to have an affect on children's answers, Order may have had an effect such that children who heard about the hierarchical group second were more likely to choose the egalitarian group when asked "Who shares more?"

ROPE Percentages

Who Was in Charge?

Parameter	ROPE_Percentage
b_Intercept	0.00000
b_Age	0.00000
b_GenderGirl	22.13125
b_Order_1Second	17.62500

A very small percentage of 'Age' posteriors fall into this region so it is likely that Age has an affect. Likewise a small percentage of the 'Order'falls into the region.

Now we can calculate and plot a probability direction score, using the posteriors

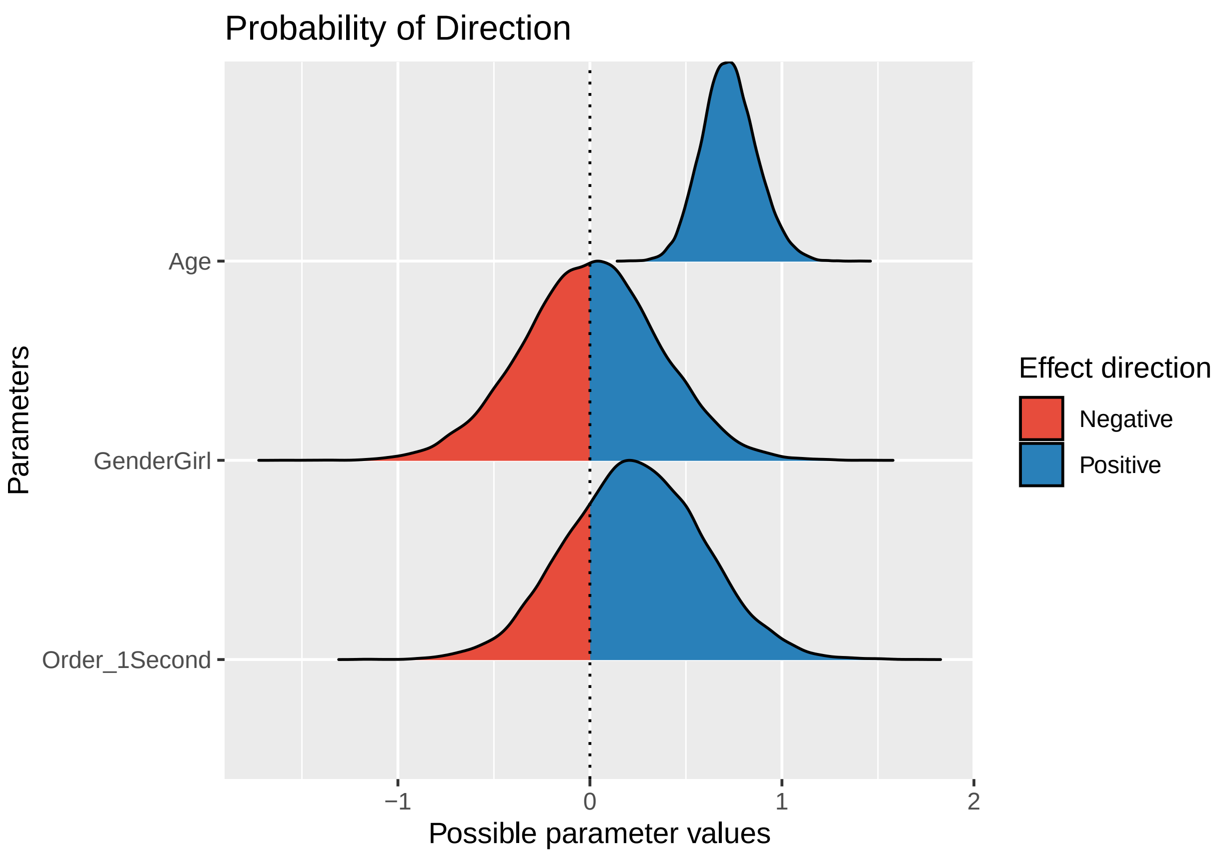

PD Percentages
Who is in Charge?

Parameter	pd
b_Intercept	100.00000
b_Age	100.00000
b_GenderGirl	50.98125
b_Order_1Second	74.63750

Here the probability of the direction is close to 100% for age and Order, but closer to 50% for Gender.

1.2.5.2 Age differences for *Who was in Charge?*

- Note, chance here is 1/3 since they chose between three characters.

Age Differences
Who Was in Charge?

Age	leader	other	total	%_Chose_leader	Bayes_Factors
4	16	26	42	38.09524	4.380123e-01

5	13	23	36	36.11111	4.155015e-01
6	27	8	35	77.14286	6.791153e+04
7	26	5	31	83.87097	6.437834e+05
8	22	4	26	84.61538	7.067786e+04

We have strong evidence that 8 year-olds chose the group without a leader more often for this question, weak evidence that 7 year-olds did so and moderate evidence that 6 year-olds did so.

1.3 Extra Analyses for Study 1

These analyses are not included in the main text, here we ask if children's answers to the test questions predict one another. First we ask whether children's answers to, 'Which ones had Someone in Charge?' and 'Who Shares More?' predicted their answers to 'Would you rather be a Wug or a Flurp?'

1.3.1 *Would you rather be a Wug or a Flurp?*

Again, fit model and check to see if the model is a 'good fit' using a posterior predictive distribution (pp_check)

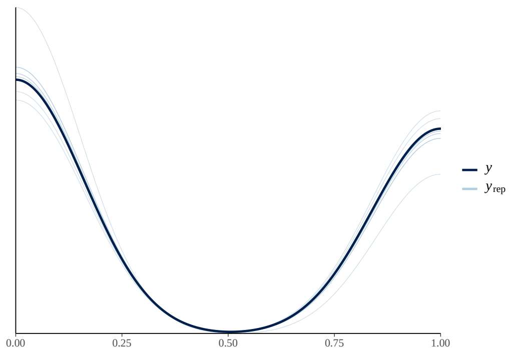

Looks pretty good (light blue lines are not deviating much from dark blue line)

1.3.1.1 ROPE and pd analyses for *Would you rather be a Wug or a Flurp?* Now we will plot the posteriors with the ROPE (region of practical equivalence). Note the ROPE was decided ahead of time, but it is somewhat arbitrary.

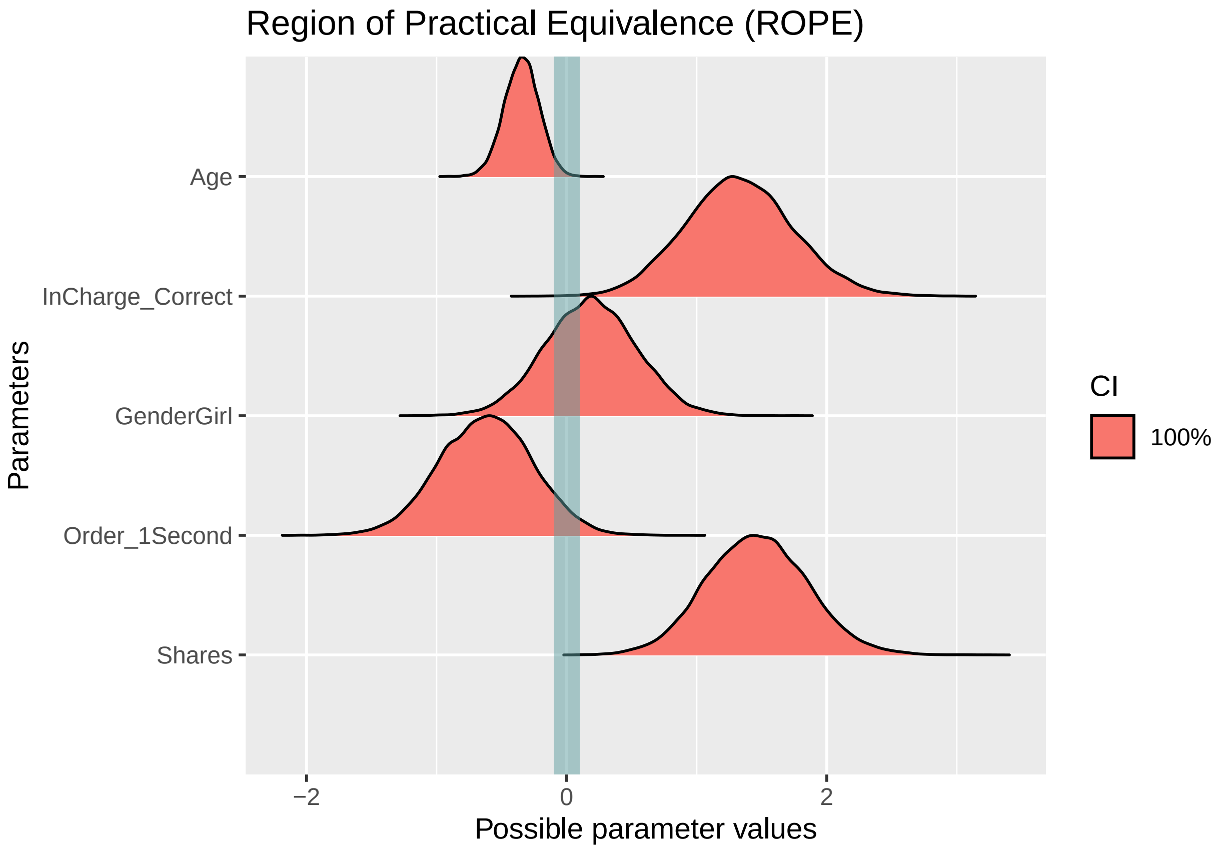

Age seems to have an affect on children's answers, Order may have had an effect such that children who heard about the hierarchical group second were more likely to choose the egalitarian group when asked "Who shares more?"

ROPE Percentages

Would you rather be a Wug or a Flurp?

Parameter	ROPE_Percentage
b_Intercept	8.21250
b_Age	3.00625
b_InCharge_Correct	0.08125
b_GenderGirl	19.49375
b_Order_1Second	5.11250
b_Shares	0.00000

A very small percentage of 'Age' posteriors fall into this region so it is likely that Age has an affect. Likewise a small percentage of the 'Order'falls into the region.

Now we can calculate and plot a probability direction score, using the posteriors

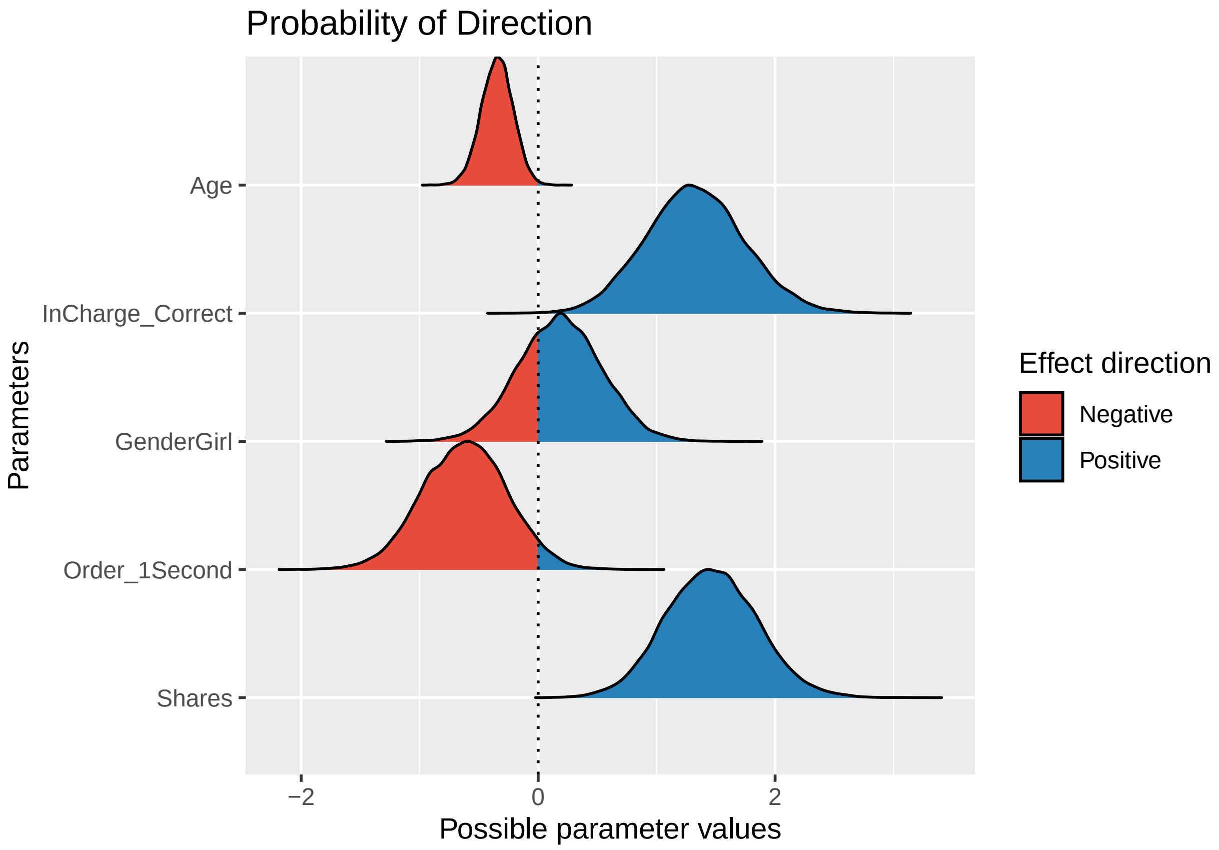

PD Percentages

Would you rather be a Wug or a Flurp?

Parameter	pd
b_Intercept	72.54375
b_Age	99.59375
b_InCharge_Correct	99.96250
b_GenderGirl	69.94375
b_Order_1Second	95.95625
b_Shares	100.00000

Here the probability of the direction is close to 100% for age and Order, but closer to 50% for Gender.

1.3.1.2 Breaking down by Factor for *Would you rather be a Wug or a Flurp?*

Did Children who correctly identify the group with a leader choose the egalitarian group more often?

Would you rather be a Wug or a Flurp?

InCharge_Correct	chose_hier	chose_egal	total	%_Chose_Hier	Bayes_Factors
0	17	34	51	33.33333	4.2369674

1	59	60	119	49.57983	0.2238339

In the above table, 0 means they got this question incorrect, 1 means they got the question correct. The children who did *not* correctly identify the leader have a stronger effect than the children who did. Neither of these Bayes Factors are strong.

Did Children's answer to 'Who Shares More' Predict Their Answers?
Would you rather be a Wug or a Flurp?

Shares	chose_hier	chose_egal	total	%_Chose_Hier	Bayes_Factors
0	35	70	105	33.33333	63.261372
1	41	24	65	63.07692	2.266787

Children's answers to 'Who Shares more?' did affect their answers to 'Would you rather be a Wug or a Flurp?'. Children who said that the egalitarian group shared more (Shares=0), were more likely to choose the egalitarian group than the children who said that the hierarhical group shares more.

1.3.2 *Who would you rather go camping with?*

Next we will ask whether children's answers to 'Who Shares More?' and 'Which ones had someone in charge?' influenced children's answers to 'Who would you rather go camping with?'

This analysis was not included in the manuscript, here we ask if children's answers predicted one another.

Again, fit model and check to see if the model is a 'good fit' using a posterior predictive distribution (pp_check)

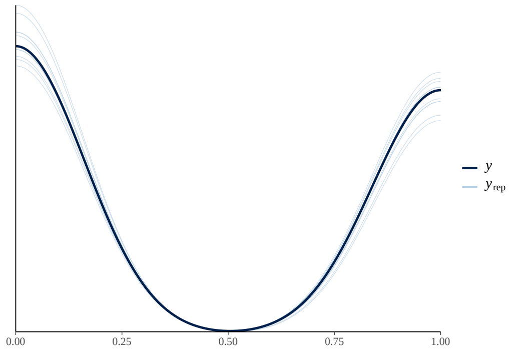

Looks pretty good (light blue lines are not deviating much from dark blue line)

1.3.2.1 ROPE and pd analyses for *Who would you rather go camping with?* Now we will plot the posteriors with the ROPE (region of practical equivalence). Note the ROPE was decided ahead of time, but it is somewhat arbitrary.

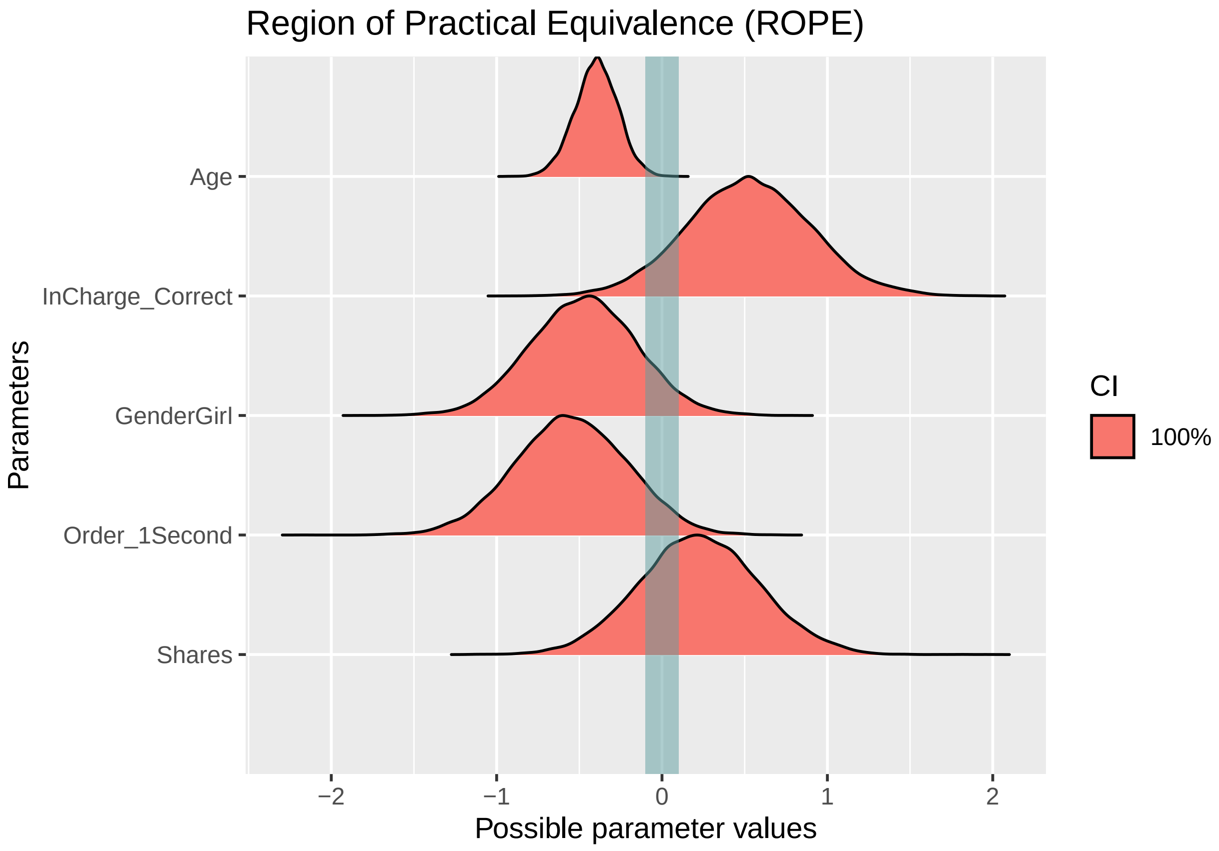

Age seems to have an affect on children's answers, Order may have had an effect such that children who heard about the hierarchical group second were more likely to choose the egalitarian group when asked "Who shares more?"

ROPE Percentages

Who would you rather go camping with?

Parameter	ROPE_Percentage
b_Intercept	0.24375
b_Age	1.03750
b_InCharge_Correct	7.81250
b_GenderGirl	8.47500
b_Order_1Second	6.76875
b_Shares	18.21875

A very small percentage of 'Age' posteriors fall into this region so it is likely that Age has an affect. Likewise a small percentage of the 'Order'falls into the region.

Now we can calculate and plot a probability direction score, using the posteriors

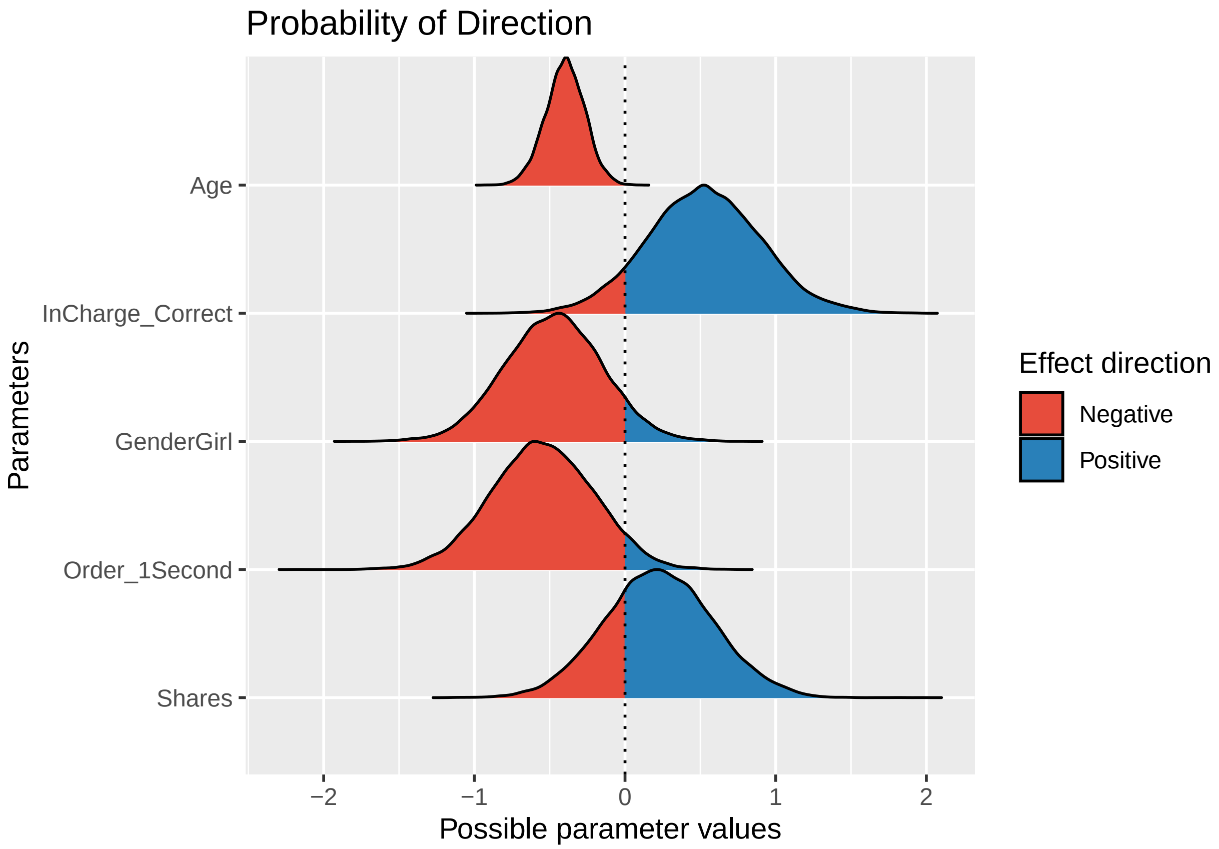

PD Percentages

Who would you rather go camping with?

Parameter	pd
b_Intercept	99.64375
b_Age	99.90625
b_InCharge_Correct	92.10625
b_GenderGirl	93.10625
b_Order_1Second	94.73750
b_Shares	73.65000

Here the probability of the direction is close to 100% for age and Order, but closer to 50% for Gender.

1.3.2.2 Breaking down by Factor for *Who would you rather go camping with?*

Did Children who correctly identify the group with a leader choose the egalitarian group more often?

Who would you rather go camping with?

InCharge_Correct	chose_hier	chose_egal	total	%_Chose_Hier	Bayes_Factors
0	22	29	51	43.13725	0.5028509

1	56	63	119	47.05882	0.2709047

In the above table, 0 means they got this question incorrect, 1 means they got the question correct. Neither group chose the hierarchical group more when asked, 'Who would you rather go camping with?'

Did Children's answer to 'Who Shares More' Predict Their Answers?
Who would you rather go camping with?

Shares	chose_hier	chose_egal	total	%_Chose_Hier	Bayes_Factors
0	45	60	105	42.85714	0.6490703
1	33	32	65	50.76923	0.2967642

Children's answers to 'Who Shares more?' did not seem to affect their answers to 'Who would you rather go camping with?'

2 Extra Analyses, Plots, and Results for Study 2

2.1 Results

2.1.1 *Who would you rather go camping with?'*

Again, we will fit the model and check to see if the model is a 'good fit' using a posterior predictive distribution (pp_check) (pp_check)

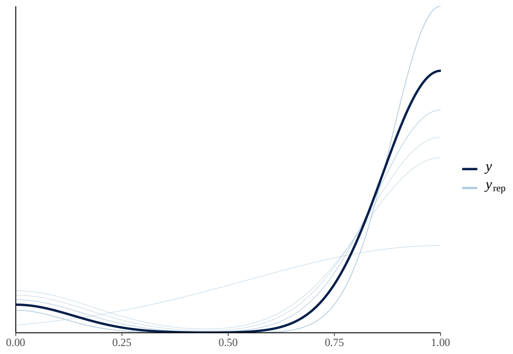

The fit isn't great. This analysis wasn't pre-registered, and this study isn't powered to look at the effects of Age etc. on children's answers. So this isn't surprising!

2.1.1.1 ROPE and PD analysis for *Who would you rather go camping with?'*

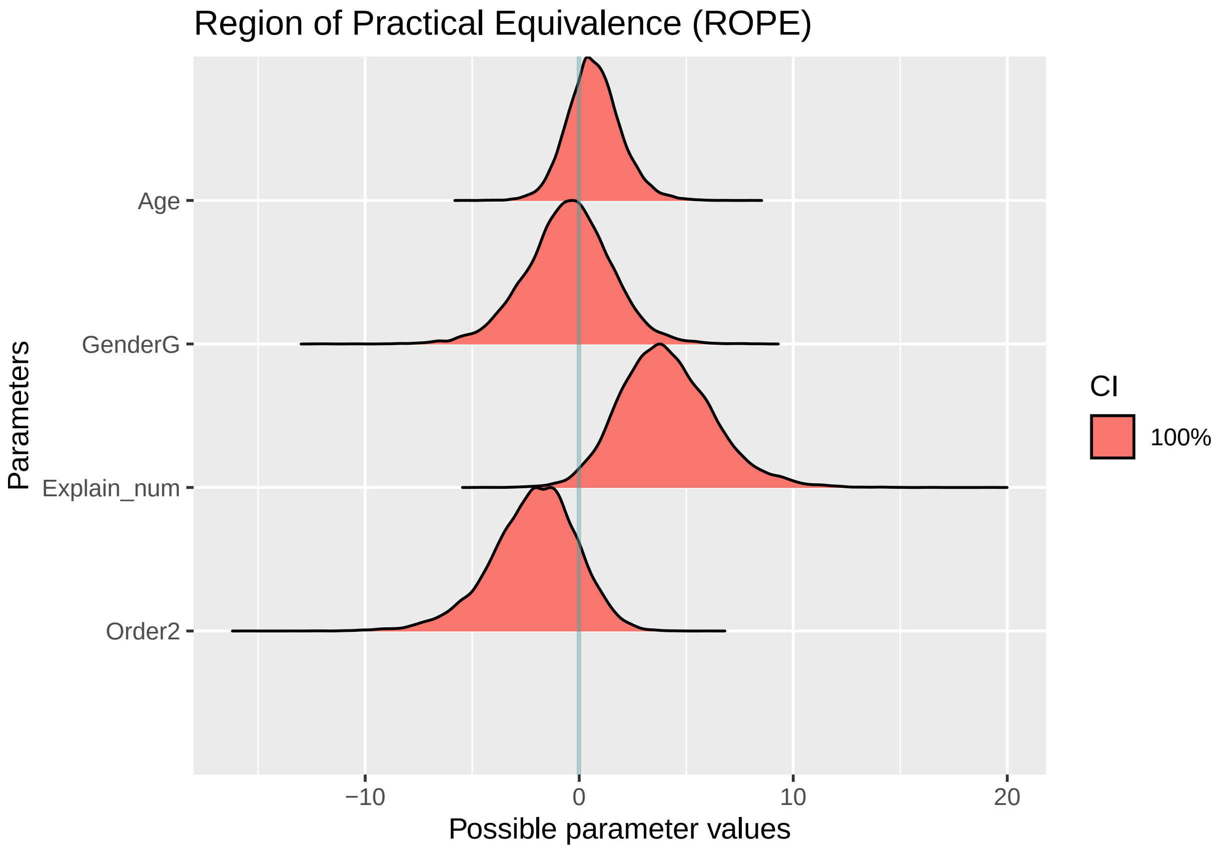

Again, this study wasn't powered to look at these effects on children's answers.

ROPE Percentages

Who Would you Rather Go Camping With?

Parameter	ROPE_Percentage
b_Intercept	0.95625
b_Age	5.65625
b_GenderG	4.18125
b_Explain_num	0.53750
b_Order2	2.58125

These plots do not provide evidence that Age, Order, or Gender had an effect on children's answers. However, it is possible that with more data these factors would affect children's answers. The most probable and plausible effect seems to be whether children said 'decision-making' when asked what was different about the two groups.

Now we can calculate and plot a probability direction score, using the posteriors

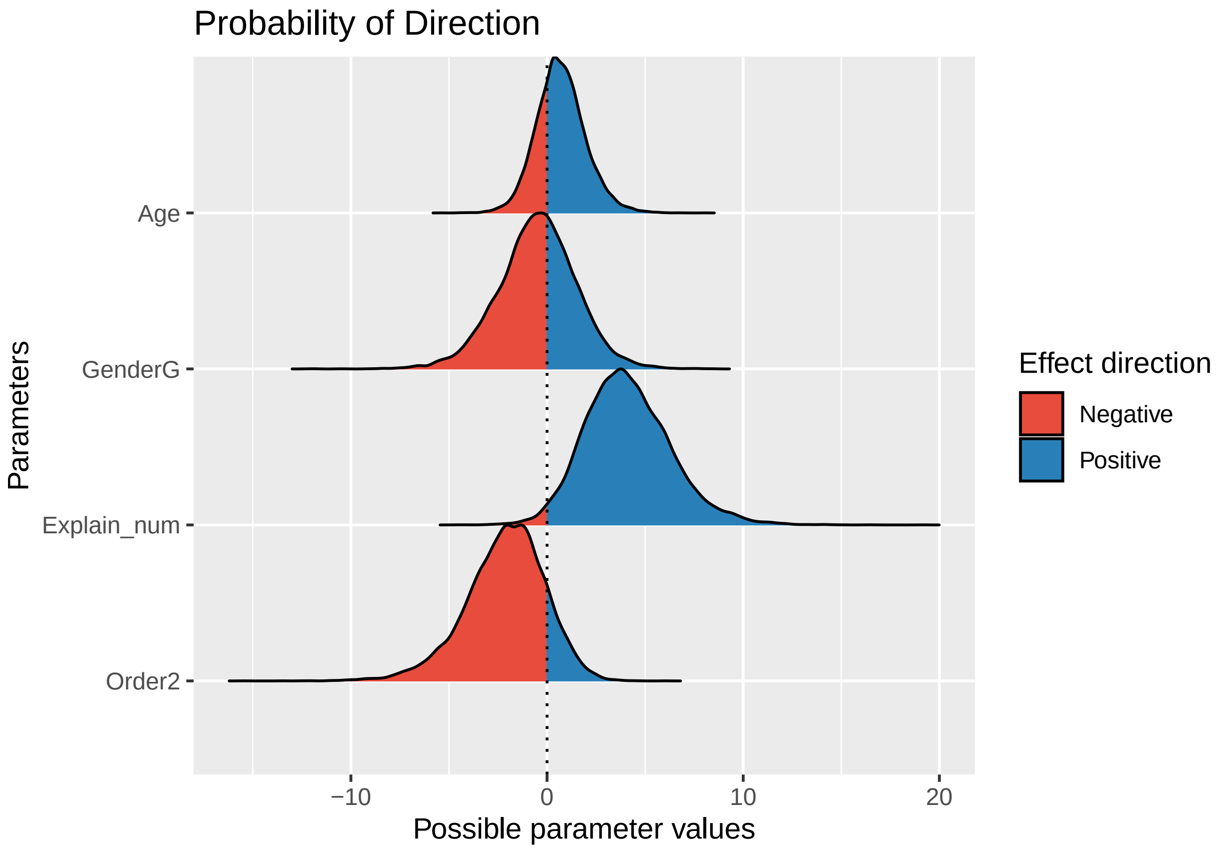

PD Percentages

Who would you rather go camping with?

Parameter	pd
b_Intercept	64.50000
b_Age	71.63750
b_GenderG	59.89375
b_Explain_num	98.19375
b_Order2	86.82500

In agreement with the posterior plots, the probability of direction is largest for whether children said decision making when we asked them what was different about the two groups. The other affects don't have strong evidence one way or another.

2.1.1.2 Age differences for *Who would you rather go camping with?'*

Age Differences

Who would you rather go camping with?

Age	chose_egal	chose_hier	total	%_Chose_Hier	Bayes_Factors
6	7	1	8	0	2.620824
7	9	2	11	NA	2.721769

8	12	0	12	NA	65.918257

This study wasn't powered to detect age differences, but we have strong evidence 8-year-olds chose the egalitarian group more often than the hierarchical group, and positive but weak evidence that 6 and 7 year olds did.

2.1.1.3 Explanation differences for *Who would you rather go camping with?'*

Explaination Differences

Who would you rather go camping with?

Explain_num	yes	no	total	%_Said Decisions	Bayes_Factors
0	3	2	5	60.00000	7.669627e-01
1	25	1	26	96.15385	1.853999e+04

Based on the very small number of children who did not include decision making in their answers, it is difficult to draw any conclusions about this.

2.1.2 *What was different about the two groups?'*

Again, fit model and check to see if the model is a 'good fit' using a posterior predictive distribution (pp_check)

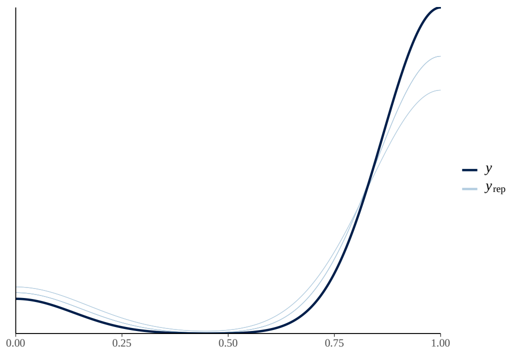

This fit doesn't look bad.

2.1.2.1 ROPE and PD analysis for *What was different about the two groups?'*

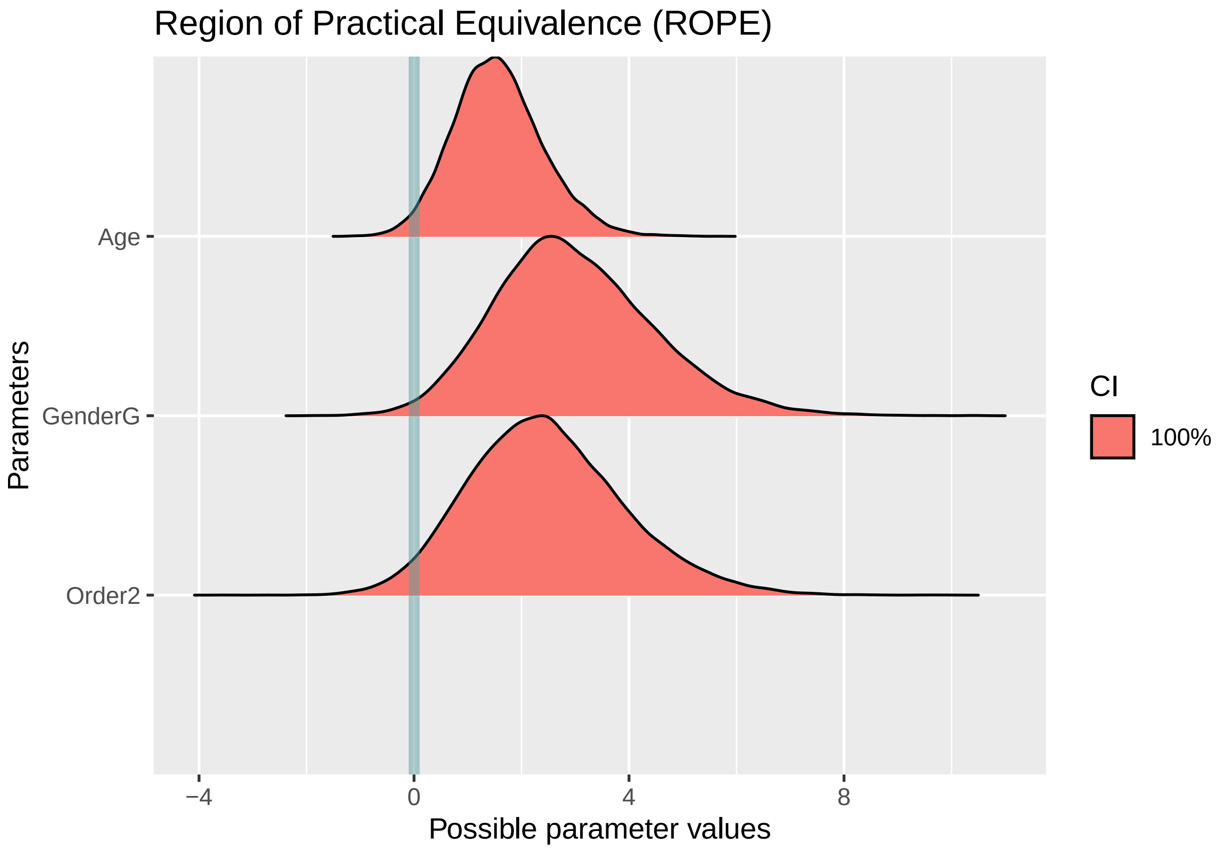

Again, this study wasn't powered to look at these effects on children's answer, but here we see evidnece that Age, Gender and Order may have affected chidlren's answers such that older children were more likley to say something about decision making, girls were more likely to say something about decision making than boys, and if they heard about the hierarchical group second, they were more likely to say that the two groups differed by decision making.

ROPE Percentages

What was different about the two groups?

Parameter	ROPE_Percentage
b_Intercept	0.13125
b_Age	1.36250
b_GenderG	0.44375
b_Order2	1.06250

Now we can calculate and plot a probability direction score, using the posteriors

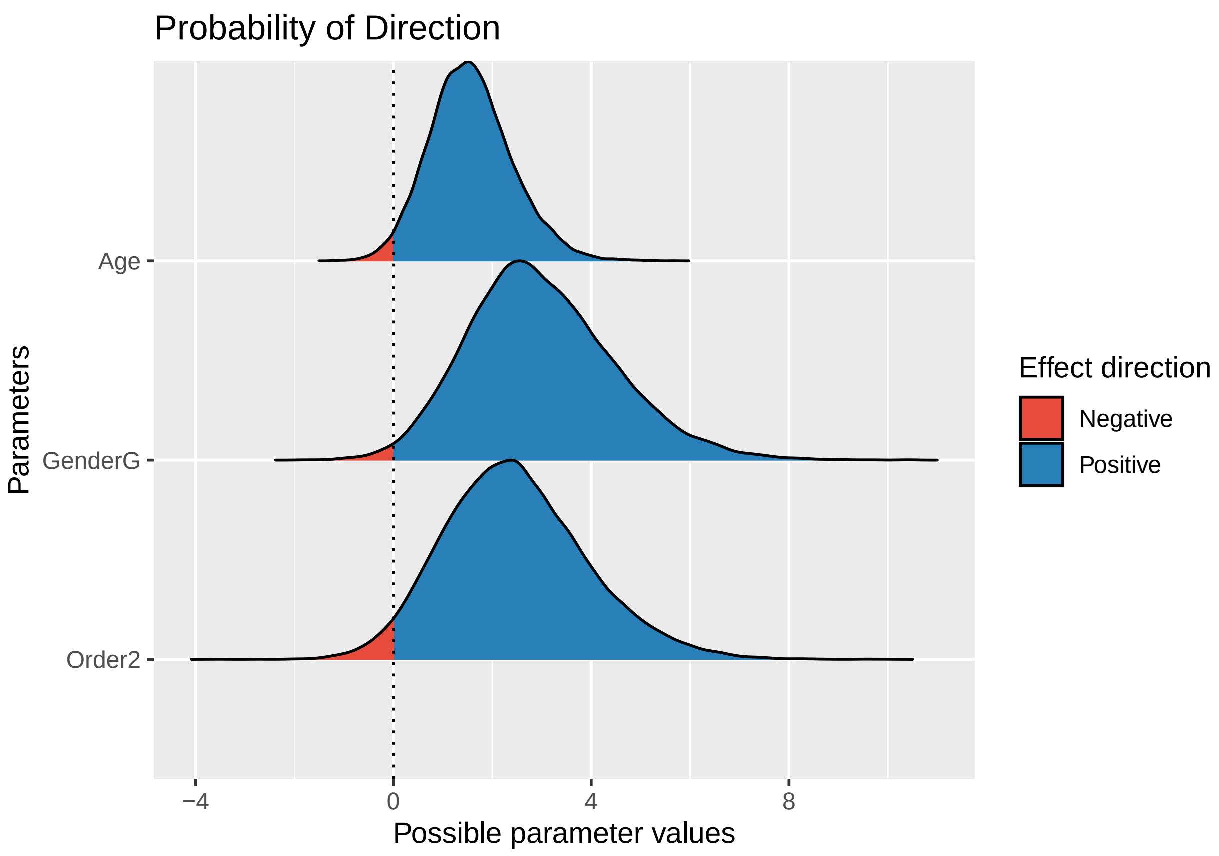

PD Percentages

What was different about the groups?

Parameter	pd
b_Intercept	98.0250
b_Age	97.9125
b_GenderG	99.0000
b_Order2	97.0250

In agreement with the posterior plots, the probability of direction is large for all the effects.

2.1.2.2 Age differences for *What was different about the two groups?'*

Age Differences

What was Different about the two groups?

Age	yes	no	total	%_Chose_Hier	Bayes_Factors
6	6	2	8	75.00000	1.178584
7	8	4	12	66.66667	0.902384
8	12	0	12	100.00000	65.918257

This experiment was not powered to detect age differences, but if we break it down by age, we only have strong evidnece that 8 year-olds reliably said that the two groups differed in their decision making. There is inconclusive evidence as to whether 6 and 7 year olds did. However these differences in Bayes Factors are difficult to interpret because of the small sample size, percentage wise, they are all trending in the right direction.

2.1.2.3 Order differences for *What was different about the two groups?'*

Order Differences

What was Different about the two groups?

Order	yes	no	total	%_Correct	Bayes_Factors
0	12	4	16	75.0	2.340547
1	14	2	16	87.5	18.573650

In the above table, the row that has 'Order'=0 means that these were the children who heard about the hierarchical group first; 1 means that children heard about the hierarchial group first. We have postive evidence that no matter whether children heard the hierarchical story first or the egalitarian story, they said something about decision making when asked, 'What was different about the two groups?'. With such a small sample, the differences in Bayes Factors are difficult to interpret.

2.1.2.4 Gender differences for *What was different about the two groups?'*

Gender Differences

What was Different about the two groups?

Gender	yes	no	total	%_Correct	Bayes_Factors
B	9	4	13	69.23077	2.340547
G	17	2	19	89.47368	18.573650

We have postive evidence that children of both genders said something about decision-making when asked 'What was different about the two groups?'. With such a small sample, the differences in Bayes Factors are difficult to interpret.

3 Supplemental Study 1 (Study S1)

In Study S1, we investigated whether younger children might prefer one of the groups if we made it easier for them to track the difference between the groups . To do so, we explicitly described the structure of the groups as we introduced them. This study was similar to Study 1 except that when we introduced the groups, we said, 'These are the Wugs. They have someone who is in charge.' or 'These are the Flurps, they do not have anyone in charge.' We did this to make the task easier for the younger children, so we could investigate whether they preferred one type of group to another.

3.1 Methods

3.1.1 Participants

We tested 86 children between the ages of 4 and 6 years old. Seven children were excluded: 3 because of experimenter error (the experimenter went off script) and 4 because the child chose to stop playing. We tested 31 four-year-olds, 20 five-year-olds, and 28 six-year-olds. Forty-one parents said their child was a boy and thirty-eight said their child was a girl. When asked to indicate racial background, 32 parents answered 'White', 31 did not answer the question, 9 answered 'Asian', 2 answered 'African American', 4 answered, 'Asian and White', 2 answered 'Asian and Native Hawaiian and White', 4 answered 'Asian and Native Hawaiian'; 2 indicated they were multiracial.

3.1.2 Procedure

The procedure was the same as in Study 1, except that when we introduced the groups we said, 'These are the Wugs. They don't have anyone in charge.' and 'These are the Flurps. They do have someone in charge'. This information was repeated throughout the story (the script can be found here: https://osf.io/s6cu9/?view_only=0f3aac3c5bdf4a05b57e7d8e4800a946

3.1.3 Results and Discussion

3.1.4 *Which ones had someone in Charge?*

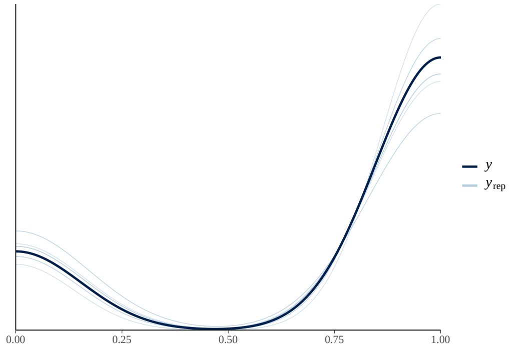

This looks pretty good (light blue lines are not deviating much from dark blue line)

3.1.4.1 ROPE and PD analysis for *Which ones had someone in charge?'* _

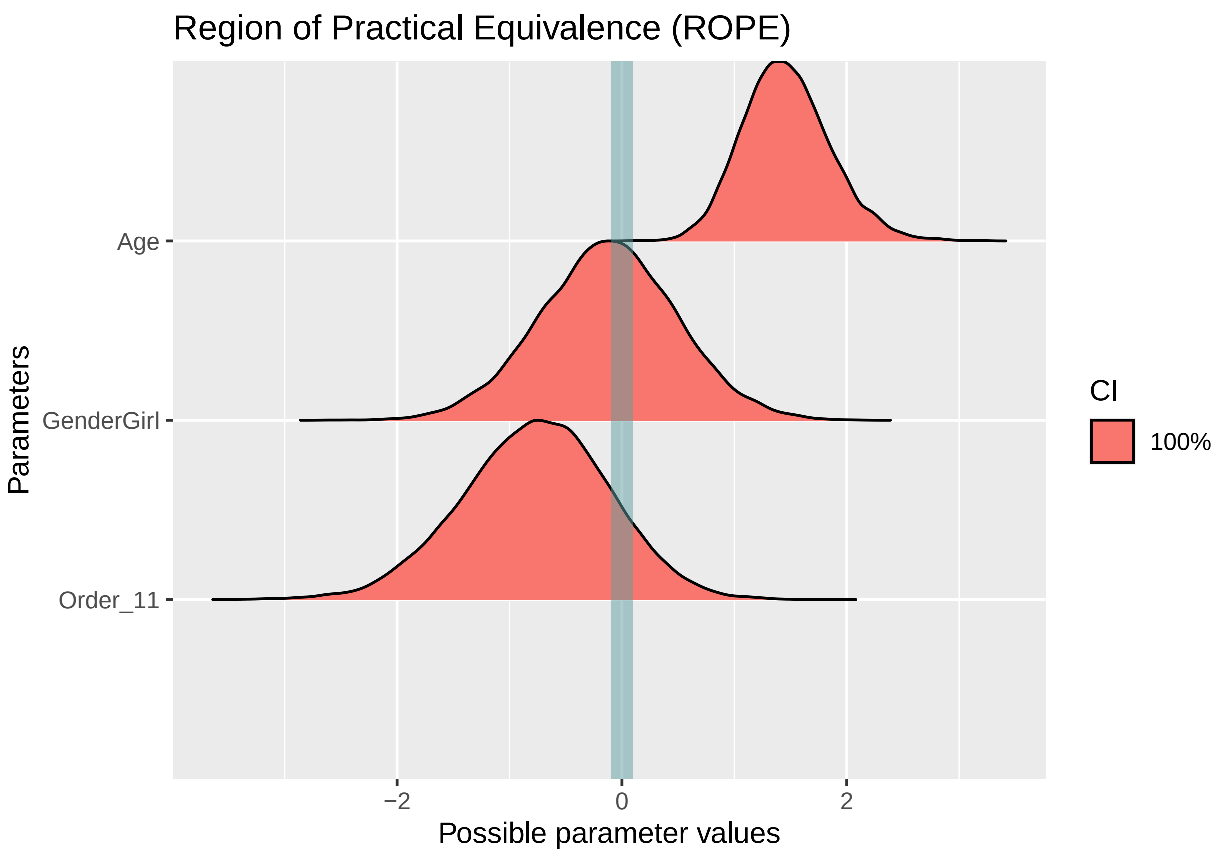

Based on these plots, and the ROPE, it is probable that Age had an effect, such that older children were more likely to choose the group with a leader (the posterior is shifted to the right of the null region). It is plausible that Order and Gender did not have an effect, since the peak of the posterior distributions lie within the null regions, but we don't have strong evidence since most of the posterior lies outside of the null region. Next we'll calculate the percent of the posterior that falls within this region.

ROPE Percentages

Which ones had someone in charge?

Parameter	ROPE_Percentage
b_Intercept	0.12500
b_Age	0.01250
b_GenderGirl	13.21875
b_Order_11	6.26875

A very small percentage of 'Age' posteriors fall into this region so it is probable that Age affected children's answers.The percentage for Gender and Order suggest there is inconclusive evidence.

Next we calculate and plot a probability direction score, using the posteriors.

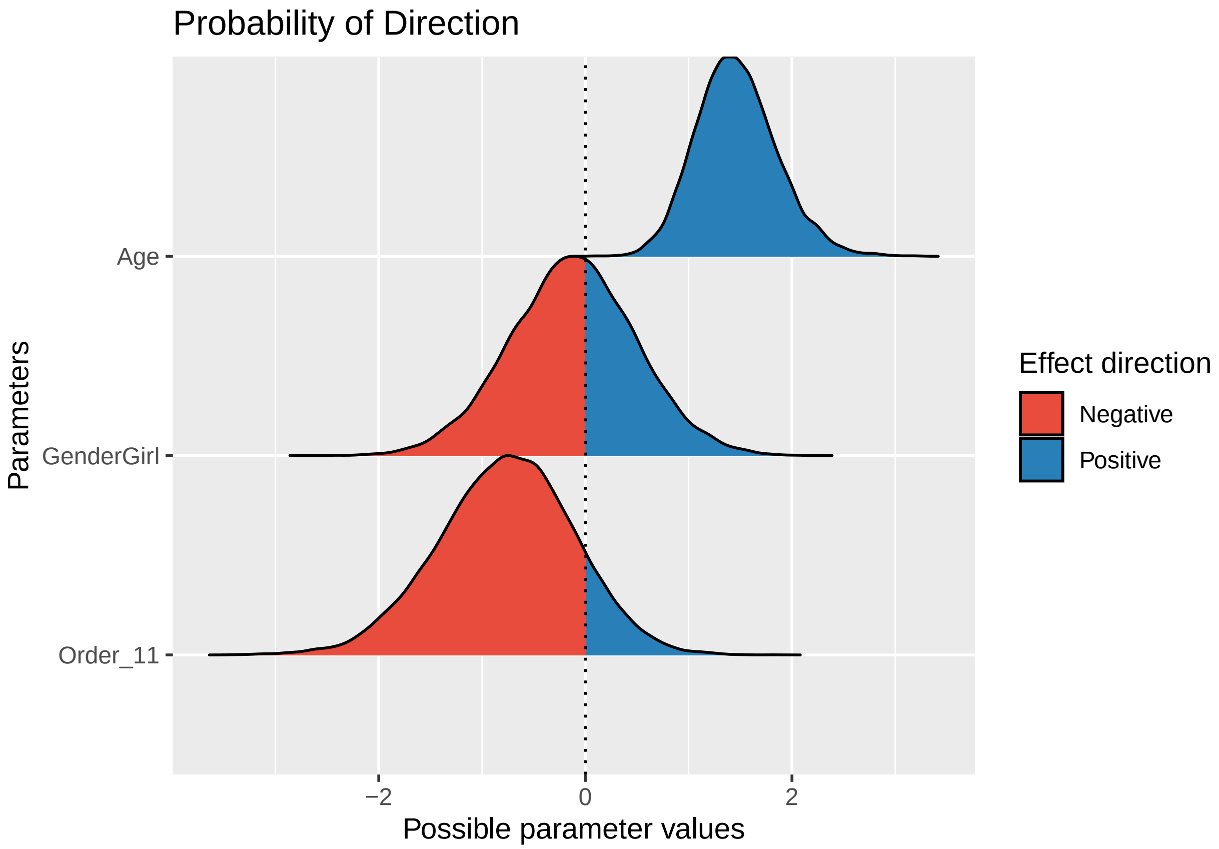

PD Percentages

Which ones had someone in charge?

Parameter	pd
b_Intercept	99.75625
b_Age	100.00000
b_GenderGirl	57.94375
b_Order_11	88.44375

The probability of the direction of effect is close to 100% for age, but 50/50 for Gender. Thus, this second analysis agrees with the ROPE analysis. Age likely affected children's answers, but not Gender, and possibly Order.

3.1.4.2 Age differences for *Which ones had Somoene in Charge?*

- Since Age had an effect, we'll look at how each age group answered this question. Seperating the children by year.

Age Differences

Which ones had Someone In Charge?

Age	chose_hier	chose_egal	total	Bayes_Factors
4	19	12	31	7.841638e-01
5	17	3	20	2.811065e+01
6	28	0	28	7.502865e+05

We have strong evidence that 5 and 6 year olds identified the group with the leader correctly.

3.1.5 *Who would you rather go camping with?*

Again, fit model and check to see if the model is a 'good fit' using a posterior predictive distribution (pp_check)

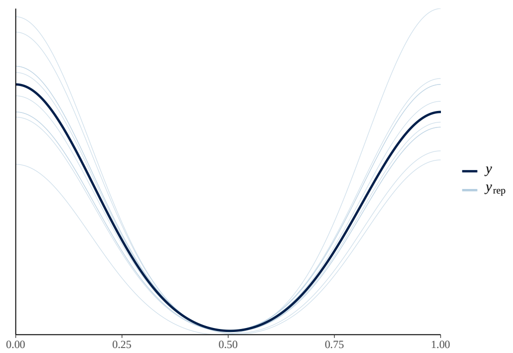

Looks pretty good (light blue lines are not deviating much from dark blue line)

3.1.5.1 ROPE and PD analysis for *Who would your rather go camping with?'*

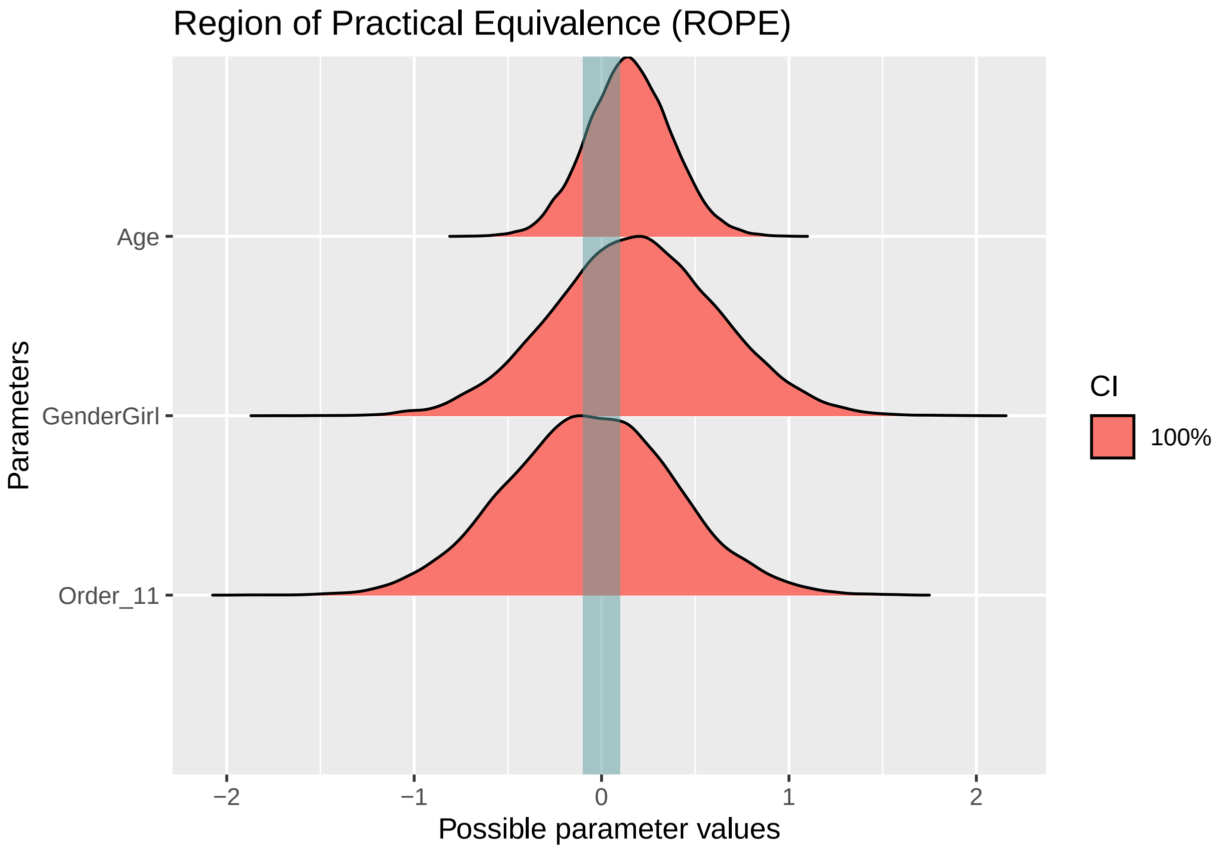

These plots suggest than none of the factors influenced children's answers to this question.

ROPE Percentages

Who would your rather go camping with?

Parameter	ROPE_Percentage
b_Intercept	5.216667
b_Age	28.516667
b_GenderGirl	16.908333
b_Order_11	16.983333

We have inconclusive evidence for each of the factors.

Now we can calculate and plot a probability direction score, using the posteriors

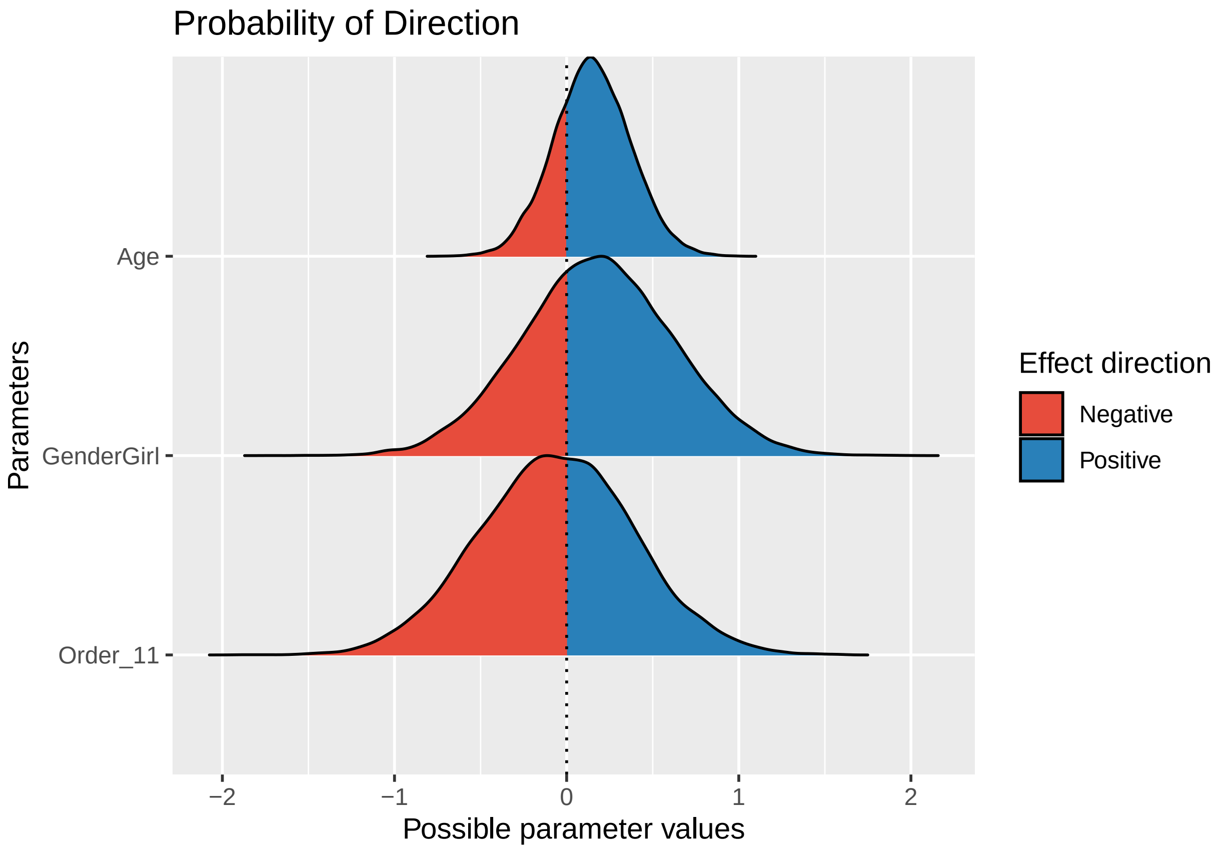

PD Percentages

Who would you rather go camping with?

Parameter	pd
b_Intercept	76.71667
b_Age	74.70417
b_GenderGirl	65.86667
b_Order_11	54.70000

This matches with the ROPE analyses, most of the percentages are too close to 50% to draw any conclusions.

3.1.5.2 Age differences for *Who would you rather go camping with?'*

Age Differences

Who Would You Rather Go Camping With?

Age	chose_hier	chose_egal	total	Bayes_Factors
4	14	17	31	0.4572707
5	8	12	20	0.6537865
6	15	13	28	0.4477781

We have inconclusive evidence for all age groups about whether they prefer one group or another.

3.1.6 *Would you rather be a Wug or a Flurp'*

Again, we first fit model and check to see if the model is a 'good fit' using a posterior predictive distribution (pp_check)

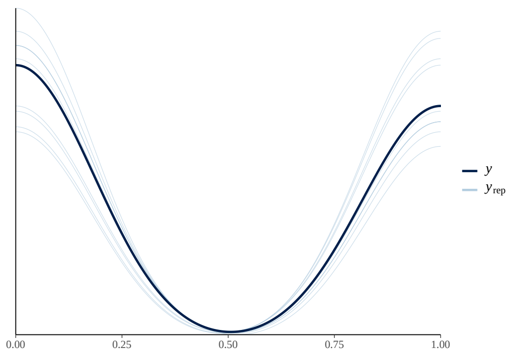

Looks pretty good (light blue lines are not deviating much from dark blue line)

3.1.6.1 ROPE and PD analysis for *Would you rather be a Wug or a Flurp?'*

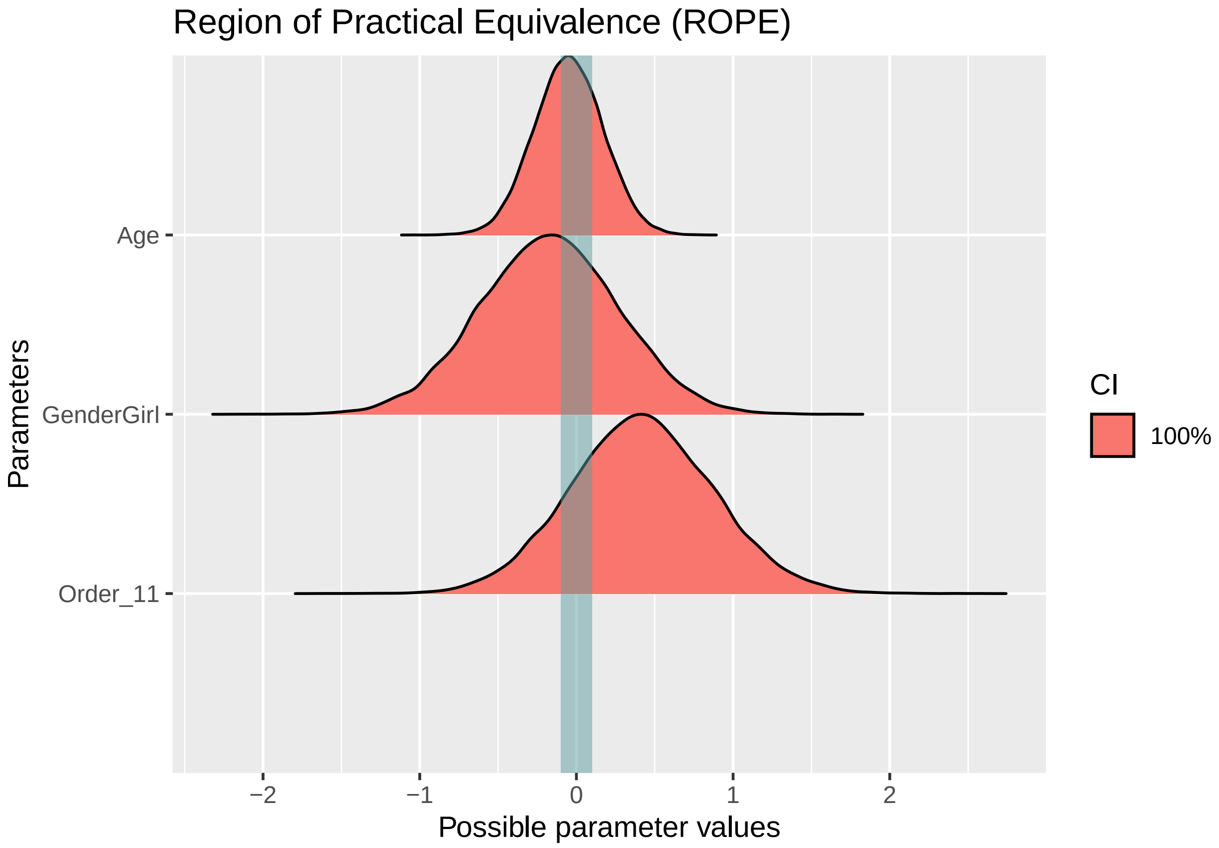

Order may have affected children's answers, but we have inconclusive evidence for the other two factors.

ROPE Percentages

Would you rather be a Wug or a Flurp?

Parameter	ROPE_Percentage
b_Intercept	6.65000
b_Age	34.28750
b_GenderGirl	16.59583
b_Order_11	11.66667

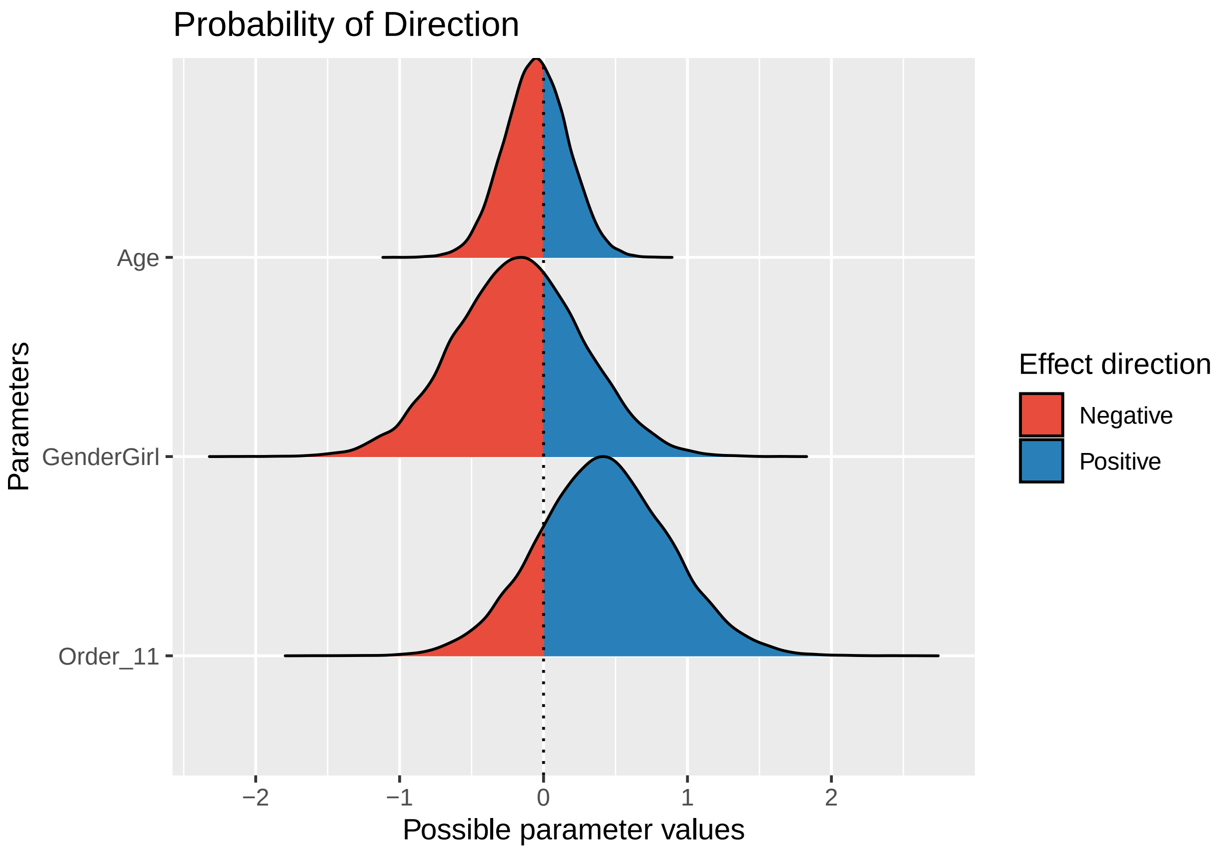

PD Percentages

Would you rather be a Wug or a Flurp?

Parameter	pd
b_Intercept	53.24583
b_Age	59.09583
b_GenderGirl	65.30000
b_Order_11	82.11667

Here the probability of the direction is close to 50% for all three factors.

3.1.6.2 Age differences for *Would you rather be a Wug or a Flurp?'*

Age Differences

Would you rather be a Wug or a Flurp?

Age	chose_hier	chose_egal	total	Bayes_Factors
4	17	14	31	0.4572707
5	9	11	20	0.5194158
6	12	16	28	0.5334244

There is inconclusive evidence as to whether any of the age groups chose the hierarhcical grop when asked if they would rather be a Wug or a Flurp.

3.1.7 *Who Shares more?'*

Again, fit model and check to see if the model is a 'good fit' using a posterior predictive distribution (pp_check)

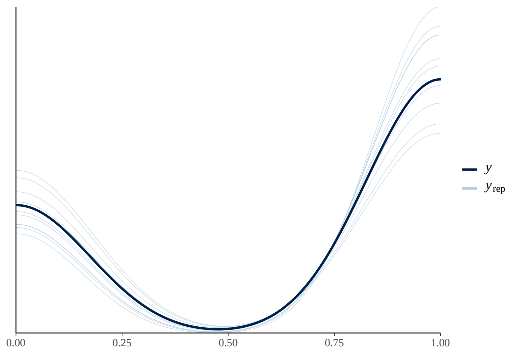

Looks pretty good (light blue lines are not deviating much from dark blue line)

3.1.7.1 Rope and PD analsysis for *Who share more?'*

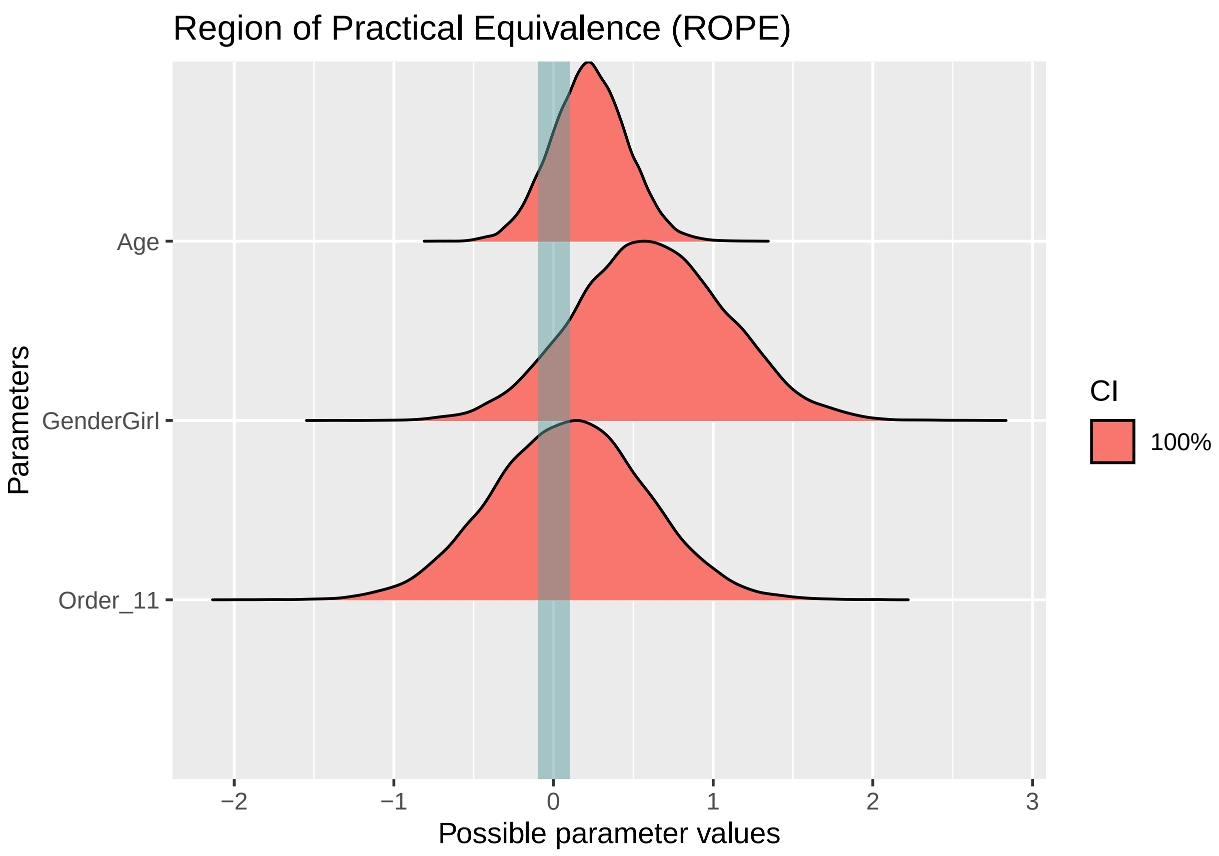

None of these plots provide strong evidnece that the factor had an effect on children's answers.

ROPE Percentages

Who Shares More?

Parameter	ROPE_Percentage
b_Intercept	5.31250
b_Age	21.09375
b_GenderGirl	7.39375
b_Order_11	16.02500

Now we can calculate and plot a probability direction score, using the posteriors

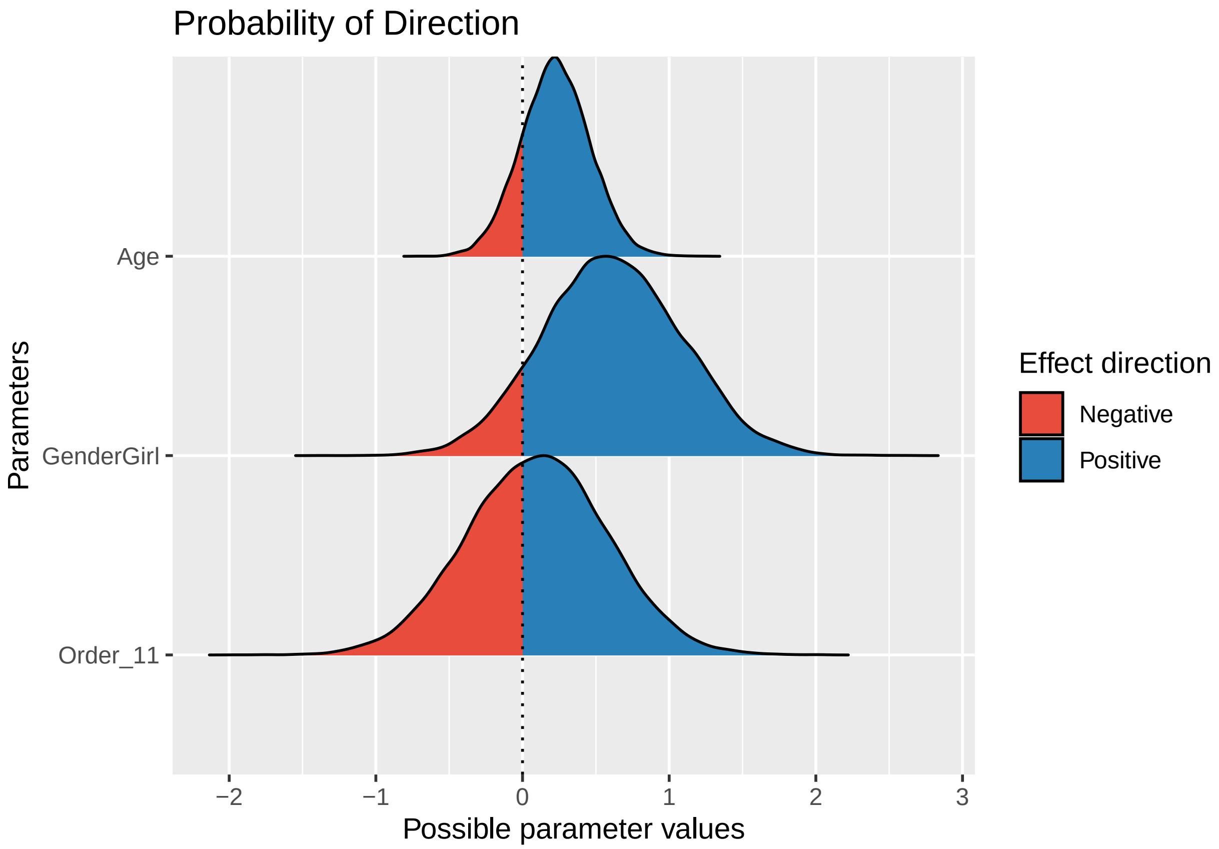

PD Percentages

Who shares more?

Parameter	pd
b_Intercept	71.56250
b_Age	83.27500
b_GenderGirl	90.40000
b_Order_11	58.33125

Gender may have had an affect.

3.1.7.2 Age differences in *Who Shares More?'*

Age Differences

Who Shares More?

Age	chose_hier	chose_egal	total	Bayes_Factors
4	10	21	31	2.1124592
5	8	12	20	0.6537865
6	8	20	28	3.6618623

We have positive evidence that 4 and 6 year olds said that the egalitarian group shares more.

3.1.7.3 Order differences for *Who shares more?'*

Order Differences

Who Shares More?

Order_1	chose_egal	chose_hier	total	Bayes_Factors
0	11	22	33	1.884517
1	15	31	46	4.127957

Children who heard about the hierarchical group first, were more likley to choose the egalitarian group when asked who shares more.

3.1.8 *Who was in Charge?*

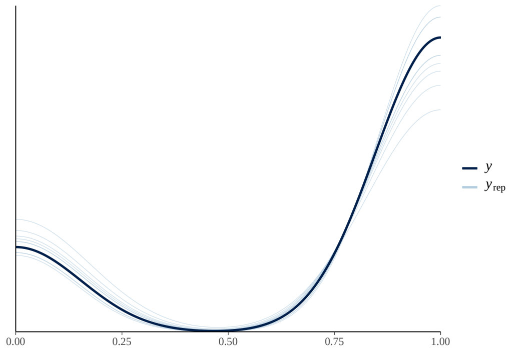

Looks pretty good (light blue lines are not deviating much from dark blue line)

3.1.8.1 ROPE and pd analyses for *Who was in Charge?* Now we will plot the posteriors with the ROPE (region of practical equivalence). Note the ROPE was decided ahead of time, but it is somewhat arbitrary.

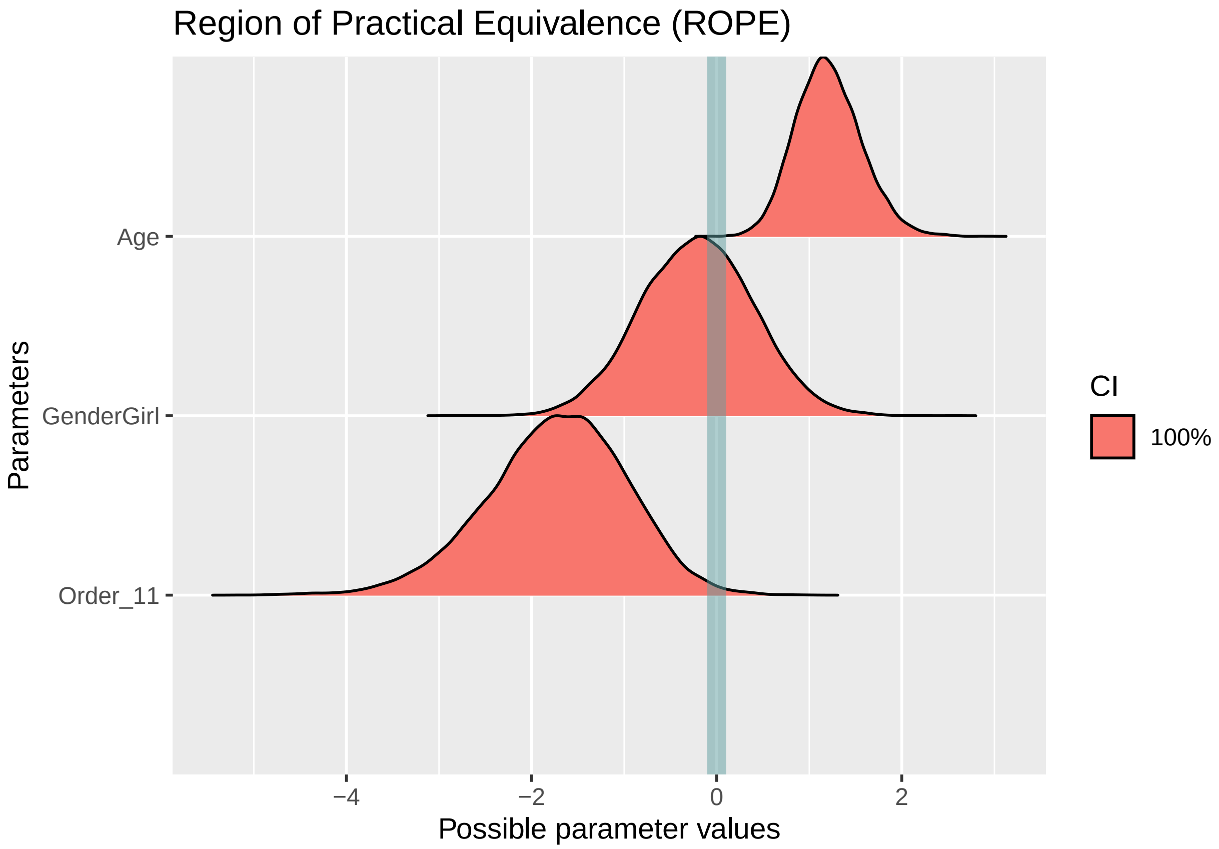

Age seems to have had an effect on children's answers, Order may have had an effect such that children who heard about the hierarchical group second were more likely to choose the egalitarian group when asked "Who shares more?"

ROPE Percentages

Who Was in Charge?

Parameter	ROPE_Percentage
b_Intercept	1.33750
b_Age	0.01875
b_GenderGirl	12.30000
b_Order_11	0.48750

A very small percentage of 'Age' and Order posteriors fall into the null region so it is likely that Age and Order have effeects.

Now we can calculate and plot a probability direction score, using the posteriors

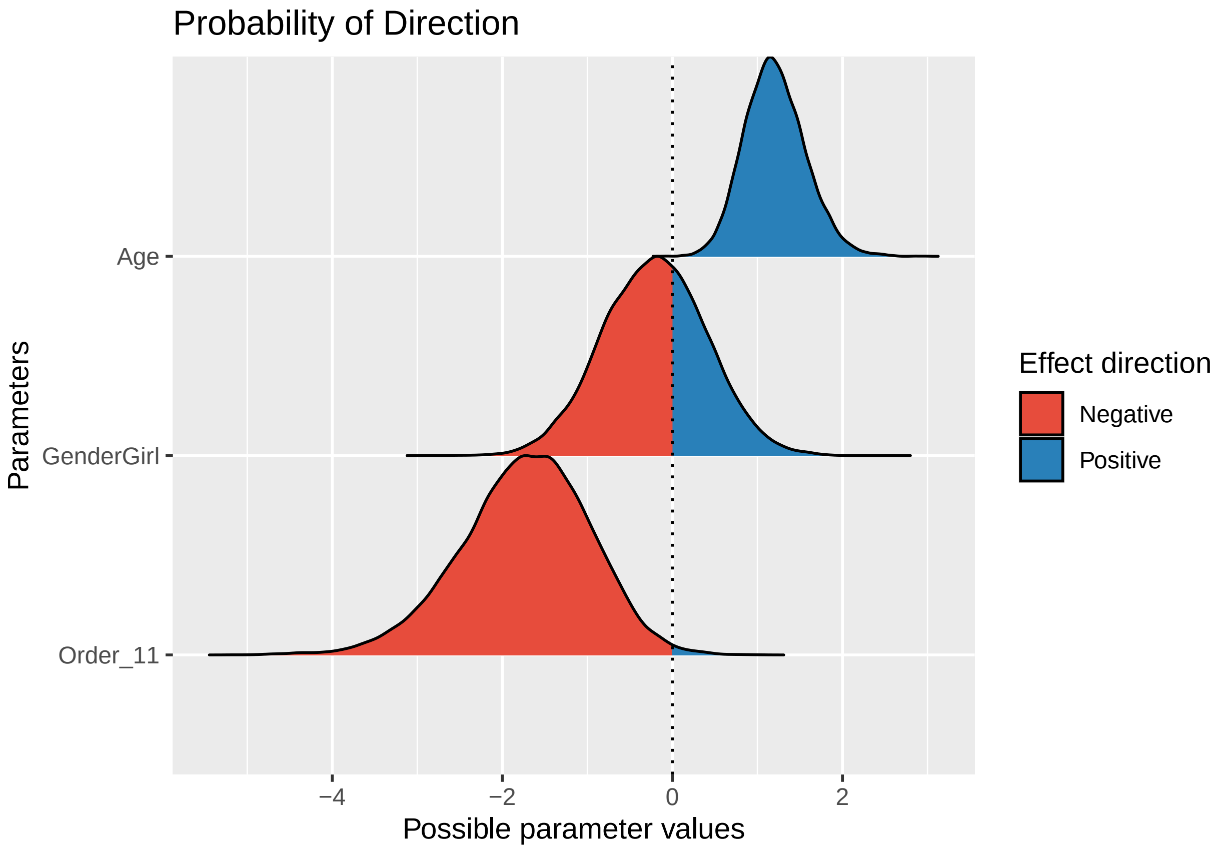

PD Percentages

Who is in Charge?

Parameter	pd
b_Intercept	95.42500
b_Age	99.98125
b_GenderGirl	63.08750
b_Order_11	99.35000

Here the probability of the direction is close to 100% for age and Order, but closer to 50% for Gender.

3.1.8.2 Age differences for *Who was in Charge?* Note, chance here is 1/3 since they chose between three characters.

Age Differences

Who Was in Charge?

Age	leader	other	total	Bayes_Factors
4	21	10	31	2.335085e+02
5	15	5	20	1.296144e+02

6	28	0	28	1.621777e+10

Here we have strong positive evidence that all of the children correctly identified the person who was in charge.

3.1.8.3 Order differences in *Who was in Charge?'*

Order Differences

Who Was in Charge?

Order_1	leader	other	total	Bayes_Factors
0	31	2	33	4.768654e+09
1	33	13	46	9.221445e+04

Unsuprisingly, when children heard about the hierarchical group second, they were more likely to correctly identify the character who was in charge. This makes sense since they were given the evidence more recently.

3.2 Discussion

These data provide mixed evidence as to whether younger children can track the structure of social groups when the group's structure is explicitly labeled. The five and six-year-olds were able to identify the group that had someone in charge (after being told); but still did not prefer either of the groups. This could be taken as evidence that younger children can distinguish between group structure, when scaffolded with explicit descriptions, but do not prefer either group. However, it is possible the children were just memorizing the phrase.
